# Identification of secondary microglial formation centers in the human fetal brain

**DOI:** 10.1084/jem.20251801

**Published:** 2026-05-18

**Authors:** Chenyun Song, Xinyu Chen, Rong Ji, Yang Liu, Yawen Han, Fangzhou Ye, Ling Zhang, Li Li, Lu Gao, Qizhi He, Lixiang Ma, Hexige Saiyin

**Affiliations:** 1 Department of Anatomy and Histology & Embryology, School of Basic Medical Sciences, Fudan University, Shanghai, China; 2 Department of Neurology, Minhang Hospital, Fudan University, Shanghai, China; 3 Department of Developmental Biology, Washington University School of Medicine, St. Louis, MO, USA; 4 Department of Anesthesiology, Huashan Hospital, Fudan University, Shanghai, China; 5 Department of Pathology, Shanghai Key Laboratory of Maternal-Fetal Medicine, Shanghai Institute of Maternal-Fetal Medicine and Gynecologic Oncology, Shanghai First Maternity and Infant Hospital, School of Medicine, Tongji University, Shanghai, China; 6 State Key Laboratory of Genetic Engineering, School of Life Sciences, Fudan University, Shanghai, China

## Abstract

Microglia migrate from the yolk sac and populate the developing brain. How microglia expand rapidly to meet the microglial demand in fast-expanding human fetal brains remains uncharted. Using thick sections in 5−22–gestational week (gw) brains and super-resolution scanning, we identified a large proliferative microglial aggregate (2.129 mm^2^) near the lateral ganglionic eminence (>12.5 gw), expanding in Down’s syndrome (DS) (4.767 mm^2^) and Edwards syndrome (ES) (3.437 mm^2^) fetal brains. Ki67^+^ microglia within the aggregates accounted for 26.65% (DS: 38.9%; ES: 46.3%) compared with 6.32% (DS: 6.01%; ES: 5.2%) in scattered microglia. This aggregate region contained a distinct microglial population characterized by the absence of phagocytic structures and complex processes, high CSF-1R expression, abundant IL-34^+^ cells, and some SPP1^+^ bipolar microglia. We termed this structure the secondary microglial formation center (SMFC). Chimeric microglia–human cortical organoids recapitulated the SMFC in an IL-34– and CSF-1R–dependent manner, indicating that the human SMFC may compensate for the microglial shortage during the fastest expansion period.

## Teaser

This study shows that a secondary microglial expansion center has been identified in fetal brains with oSVZ.

## Introduction

Originating from the yolk sac (YS), microglia serve as the primary resident phagocytes in the brain, maintaining structural and functional homeostasis throughout development and adulthood (Askew et al., 2017; Bennett et al., 2018; Monier et al., 2006; Nimmerjahn et al., 2005). In humans, microglia enter the developing brain at around 4.5−5.5 gestational weeks (gw) and eventually account for ∼15% of all adult brain cells (Herculano-Houzel, 2012; Verney et al., 2010). The human fetal brain has a large outer subventricular zone (oSVZ), which drives the large size and complexity of the human cortex (Lui et al., 2011). The oSVZ emerges at ∼13 gw, rapidly expands following its formation, and resolves around 24 gw (Baburamani et al., 2020; Hansen et al., 2010; Lui et al., 2011). However, the mechanisms by which the microglial pool robustly expands to meet the cellular demand during this oSVZ period remain poorly understood.

YS-derived microglia enter the fetal brains prior to the generation of astrocytes and oligodendrocytes and the formation of the oSVZ (Lui et al., 2011). Microglia reach the brain through circulation-dependent or circulation-independent routes in murine and zebrafish models (Ginhoux et al., 2010; Xu et al., 2016). CSF-1 receptor (CSF-1R) activation by CSF-1 and IL-34 dictates microglial homeostasis and regional distribution in the murine fetal brain (Freuchet et al., 2021; Kana et al., 2019; Wang et al., 2012). Anatomically, IL-34 regulates microglial maintenance in the murine forebrain and retina, while CSF-1 governs the murine cerebellum; interestingly, IL-34 has also been shown to inhibit microglial maturation and phagocytosis (Devlin et al., 2024, *Preprint*; Kana et al., 2019; O'Koren et al., 2019). Notably, human IL-34, an evolutionarily conserved protein, does not cross-react with murine CSF-1R, highlighting species-specific differences (Baghdadi et al., 2018; Freuchet et al., 2021). Clinically, microglial dysregulation is implicated in a wide spectrum of neurodevelopmental and adult disorders (Wilson et al., 2023), including Down’s syndrome (DS) (Jin et al., 2022), obsessive–compulsive disorder (Frick and Pittenger, 2016), Rett syndrome (Maezawa and Jin, 2010), and Huntington’s disease (Yang et al., 2017). However, the precise roles of IL-34 and CSF-1 in human brain microglia remain elusive due to the scarcity of primary human samples and the lack of robustly validated in vitro models.

Brain organoids derived from human induced pluripotent stem cells (hiPSCs) recapitulate key anatomical features of the early human fetal brain, especially the oSVZ, serving as an invaluable tool for studying human-specific neurodevelopment (Di Lullo and Kriegstein, 2017; Lancaster et al., 2013; Zhou et al., 2024). However, conventional brain organoids lack YS-derived microglia (Lancaster et al., 2013). Incorporating human microglia into these organoids partially mimics their in vivo physiological roles and complex morphology, while simultaneously enhancing overall organoid maturation (Ormel et al., 2018; Park et al., 2023; Zhang et al., 2023). Therefore, microglia-integrated chimeric human brain organoids offer a robust platform to study the developmental dynamics of microglia in the human fetal brain.

Herein, we combined immunostaining with multiple antibodies and state-of-the-art high-resolution scanning of thick (50 µm) sections to map the precise structural and spatial information of human fetal brain microglia, including healthy and diseased brains. We subsequently utilized chimeric microglia–brain organoids to replicate the precise colonization patterns observed in vivo. In the 12.5–22-gw fetal brains, we identified a large microglial expansion center with distinct morphology, specific anatomical locations, high proliferative microglia, and high-density IL-34^+^ cells, a structure we term the secondary microglial formation center (SMFC). Notably, the emergence of the SMFC coincides with oSVZ formation, and this structure is significantly expanded in the fetal brains of DS and Edwards syndrome (ES). Furthermore, we successfully recapitulated the SMFC in chimeric microglia–human cortical organoids (hCOs). Together, these findings highlight the critical developmental and clinical significance of the SMFC in human brain expansion.

## Results

### The precise structural and spatial details of intact microglia in early fetal brains

**Figure S1.**
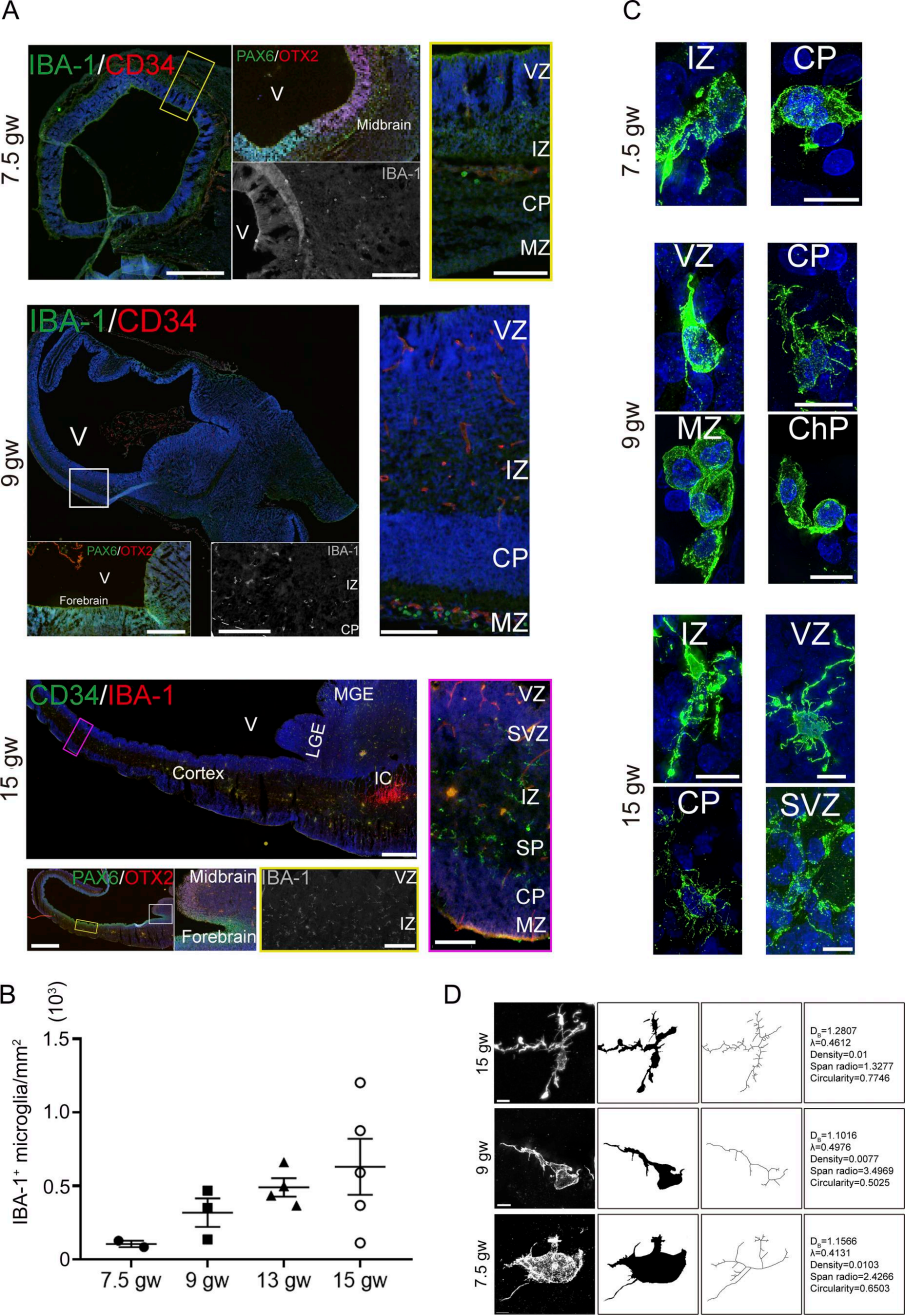
**Changes of microglial density and complexity in fetal brains (related to Fig. 1). (A)** Whole brain sections immunostained with CD34 and IBA-1 antibodies in the fetal brain at 7.5, 9, and 15 gw (the left in upper panel and the upper left in middle and bottom panels). Immunostaining of PAX6 and OTX2 antibodies delineated the forebrain and midbrain (the middle in the upper panel; the lower left in the middle and the bottom panels). The gray panels are the IBA-1 signal. Right, the magnified image (boxed, left). IBA-1^+^ microglia are predominantly localized in the IZ and SP, with a limited presence in the SVZ and a few in the MZ/CP region. Scale bars (7.5 and 9 gw), 400 µm (left), 200 µm (middle), and 100 µm (right). Scale bars (15 gw), 2 mm (left, whole slides) and 100 µm (gray channel [IBA-1] and right). **(B)** Microglial density in the fetal brains at 7.5, 9, 13, and 15 gw (fetuses, *n* = 7; the data of whole slides). **(C)** Super-resolution images of microglia in the cortical region of fetal brains at 7.5, 9, and 15 gw; recording, SIM. Scale bars, 10 µm. **(D)** Structural analysis of microglia in different gw fetal brains shows an increase in microglia’s complexity following the rise of gw. SP, subplate; IZ, intermediate zone; MZ, mantle zone.

The size of human microglia is ∼50 µm (Torres-Platas et al., 2014). Therefore, immunostaining in thin brain sections (<20 µm) makes it impossible to visualize intact microglia. To see the spatiotemporal status of intact microglia in the human fetal brain, we used IBA-1, CD34, and Ki67, or IBA-1, Ki67, and vimentin antibody immunostaining in 50-µm human fetal brain sections (5−22 gw) (Table S1). We scanned the sections using high-resolution confocal microscopy, including structured illumination microscopy (SIM) with a resolution of near 64 nm. Consistent with previous reports (Menassa et al., 2022; Rezaie and Male, 1999; Verney et al., 2010), a limited number of microglia with amoeboid or bipolar morphology were distributed in the early cortex of 7.5 gw fetal brain, and this density increased with advancing gestational age (Fig. S1, A and B). In SIM images, microglia were amoeboid at the early stage (7.5 gw); subsequently, microglial processes became more complex and branched, and morphological complexity scores increased from 7.5 to 15 gw (Fig. S1, C and D).

**Figure 1.**
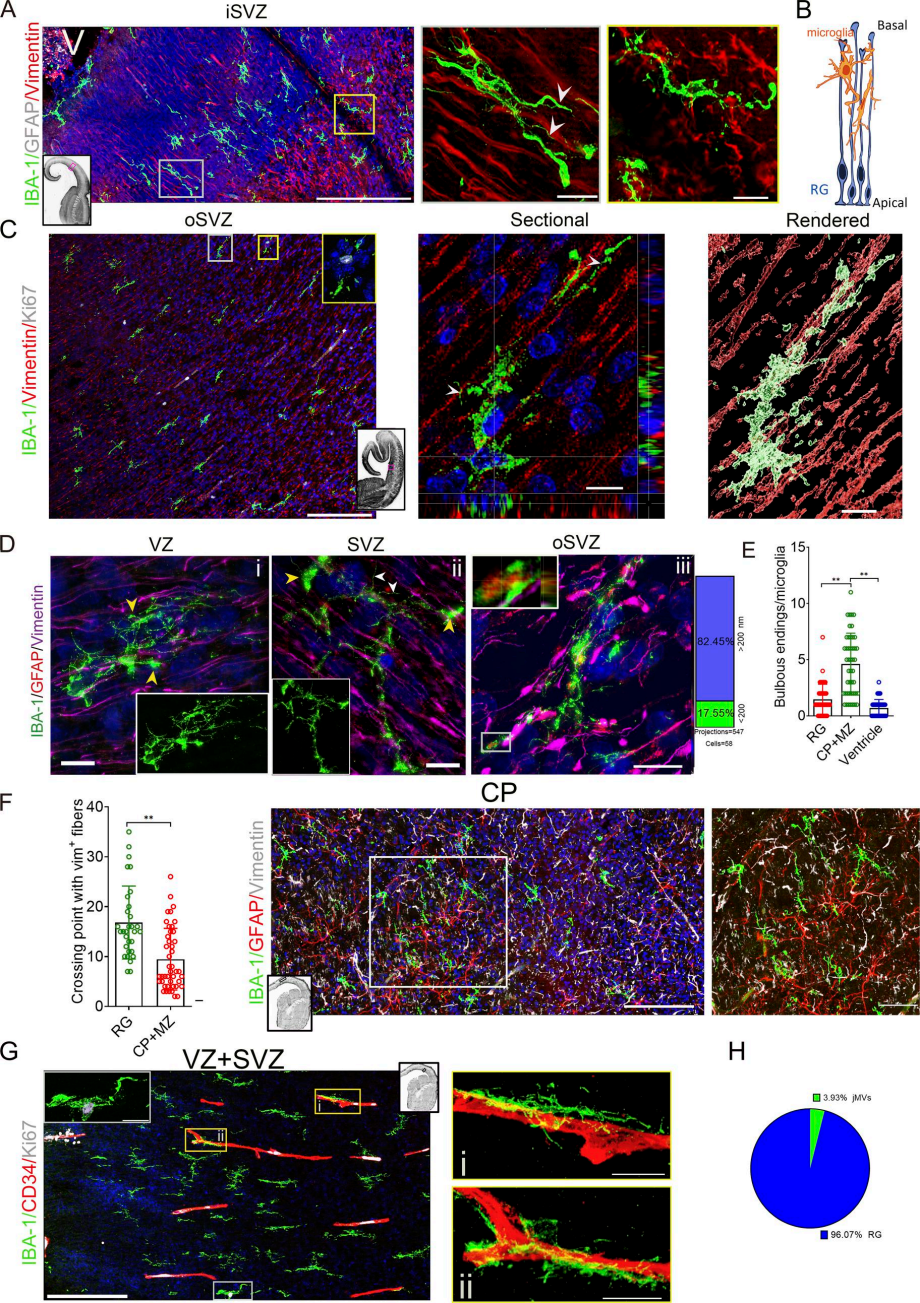
**Precise structural and spatial details of intact microglia in the early fetal brain. (A and B)** Immunostaining of the fetal brain with IBA-1, GFAP, and vimentin antibodies in iSVZ and VZ shows that the microglia nested between the RG fibers and their processes intensively interacted with the vimentin^+^ RG fibers (middle and right panel, the boxed region [yellow and white]; white arrowheads, the contact of the microglial projections with the vimentin^+^ fibers) (A). Panel B shows schematic illustrating the interaction between microglia and RG fibers. V, ventricle. Scale bars, 200 µm (left) and 20 µm (middle, right). **(C)** Immunostaining of 13-gw fetal brain sections with IBA-1, Ki67, and vimentin antibodies in the oSVZ region revealed the orientation of the main body axis of microglia and vimentin^+^ RG fibers. Most RG scaffolding microglia are Ki67^−^. Middle, the sectional view; left, 3D rendering of the Z-stack image (yellow rectangle, Ki67^+^ microglia in the insert; white arrowheads, contact of microglial projections with vimentin^+^ fibers). Scale bars, 200 µm (left) and 20 µm (middle, right). **(D)** 3D high-resolution image of immunostaining with IBA-1, GFAP, and vimentin antibodies in VZ (i), SVZ (ii), and oSVZ (iii) (white arrowheads, the projections with <200 nm in diameter; yellow arrowheads, bulbous endings). The inner inserts in i and ii, IBA-1 immunostaining. The insert in iii, a GFAP^+^ fragment in a bulbous ending of microglia. The right graph shows the percentage of the projections with <200 nm. Scale bars, 10 µm. **(E)** Counting bulbous endings in RG, CP, and ventricle microglia (fetuses, *n* = 4; data, mean ± SD; each dot, microglia; one-way ANOVA with Holm–Sidak’s multiple comparisons test, **P < 0.01). **(F)** Counting the microglial processes crossed with vimentin^+^ RG fibers in SVZ + VZ (RG), CP + MZ (fetuses, *n* = 4; each dot, microglia; data, mean ± SD; Mann–Whitney *U* test, **P < 0.01). Middle, the immunostaining with IBA-1, vimentin, and GFAP antibodies in the CP + MZ region of a 13-gw fetal brain. Rectangle, the magnified region on the right. Lower panel (black and white), the whole view of the brain section. Inner black rectangle, the region of the image. Scale bars, 200 µm (left) and 50 µm (right). **(G)** IBA-1, CD34, and Ki67 antibody immunostaining in VZ and SVZ of a 13-gw fetal brain. A few microglia interacted with the microvessels, and Ki67^+^/IBA-1^+^ cells are rare. Rectangles are the magnified images on the right. Upper right (black and white), the whole view of the brain section. Inner black rectangle, the location of the image. Scale bars, 200 µm (left) and 20 µm (right). **(H)** Percentage of RG and microvasculature-interacting microglia in human fetal brains (fetuses, *n* = 4; total images, *n* = 15).

Microglia in the ventricular zone (VZ) and subventricular zone (SVZ), including the inner SVZ (iSVZ) and oSVZ, nested between the vimentin^+^ radial glial (RG) fibers, and the major body axis of the microglia aligned parallel to the orientation of the RG fibers (Fig. 1, A–C); microglial processes interacted with RG fibers (Fig. 1, A and C). SIM images revealed that the microglia, which interact with GFAP^+^/vimentin^+^ fibers, had multiple bulbous endings (Fig. 1 D i–iii), a microglial phagocytic unit (Davalos et al., 2005; Nimmerjahn et al., 2005). Notably, some large microglia (>80 µm) exhibited several thick projections with bulbous endings, some of which contain phagocytic GFAP^+^ fragments, and enormous thin projections (<200 nm in diameter), which represent 17.6% (96/547; cells = 58) of all projections, and cross with vimentin^+^ fibers (Fig. 1 D i–iii). The cortical plate (CP) microglia display more bulbous endings than the RG-attaching or ventricular microglia (Fig. 1 E), and the projections of RG-attaching microglia had more crossing points with the RG than those in the CP regions (Fig. 1 F). Approximately 4% of the microglia in the iSVZ and oSVZ interact with microvasculature (Fig. 1, G and H). Together, this approach provides precise structural, physiological, and spatial details about intact microglia, especially the structures smaller than 200 nm and physiology-related signatures, such as bulbous endings and scaffolding.

### Highly proliferative microglia aggregate with a unique morphology present in the fetal brain with the oSVZ

**Figure 2.**
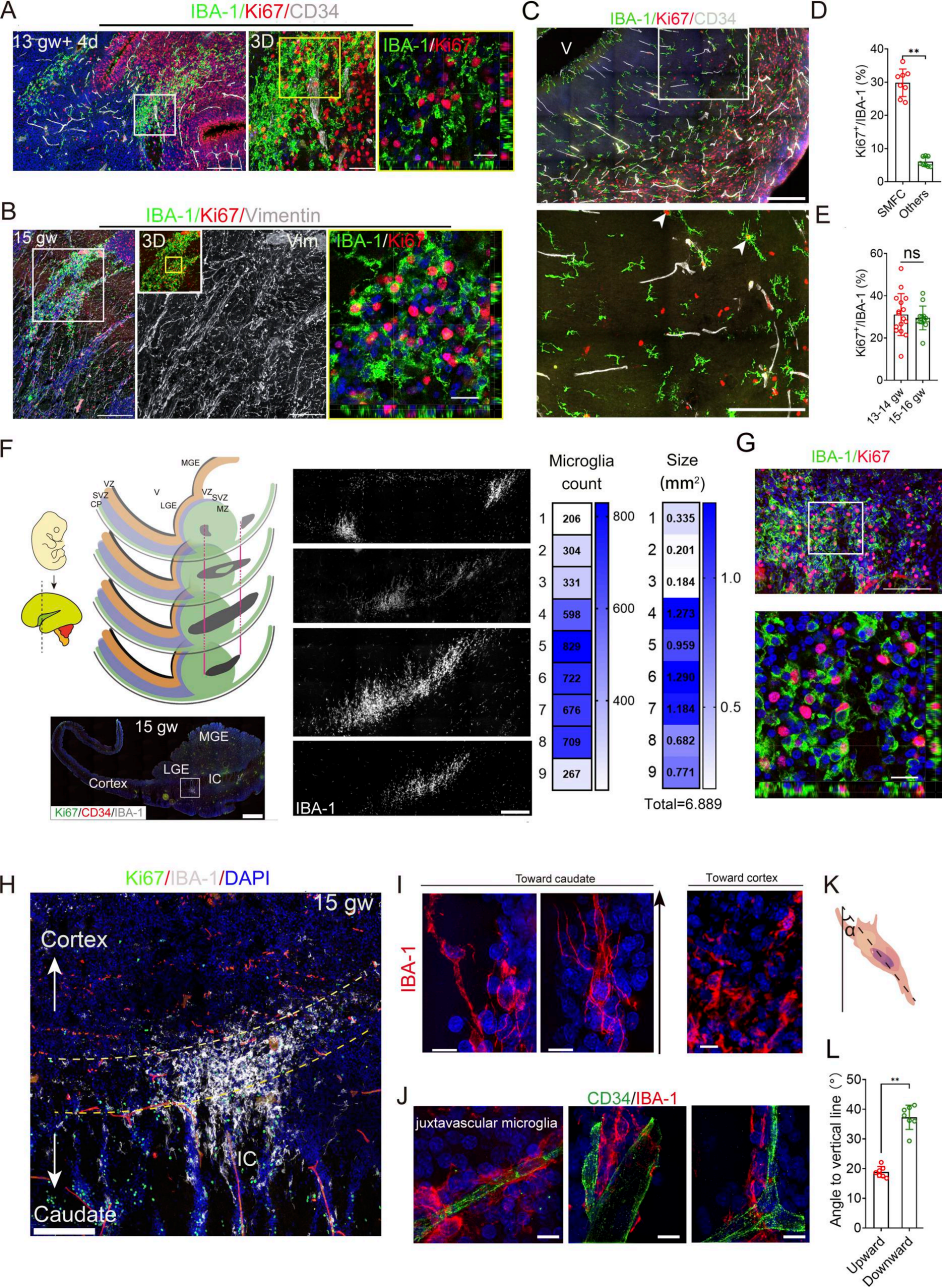
**Identification of proliferative microglial aggregates in the fetal brain. (A and B)** 3D stitching images of IBA-1, Ki67, and CD34 (A) or vimentin (B) antibody immunostaining in two fetal brains (13−15 gw) revealed large microglial aggregates with abundant Ki67^+^ microglia. Middle, the orientation of vimentin^+^ fibers in the center of the microglial aggregates with a D insert (B). Right, magnified orthogonal views (A and B). Scale bars (A), 400 µm (left), 50 µm (middle), and 20 µm (right); scale bars (B), 400 µm (left), 100 µm (middle), and 20 µm (right). **(C)** 3D stitching images of IBA-1, Ki67, and CD34 antibody immunostaining in the cortical layers of 13-gw fetal brains. White arrowheads, IBA-1^+^/Ki67^+^ cells. Scale bars, 200 µm (top) and 100 µm (bottom). **(D)** Percentage of Ki67^+^ microglia in the microglial aggregate and scattered microglia in other regions (fetuses, *n* = 8; data, mean ± SD; Mann–Whitney *U* test; dot, one brain; **P < 0.01). **(E)** Percentage of Ki67^+^ microglia in human fetal brain samples at 13−14 gw and 15−16 gw (fetuses, *n* = 6; mean ± SD; Mann–Whitney *U* test; ns, nonsignificant; dot, a section). **(F)** Illustration on the left shows sectional positions. The lower left shows a whole view of the fetal brain section. MGE, medial ganglionic eminence; V, ventricle; MZ, mantle zone. The middle shows the entire view of the microglial aggregate across four consecutive sections. The right two graphs show the number of IBA-1^+^ microglia and size of microglial aggregate in nine consecutive sections of a 15-gw fetal brain. Scale bars, 2 mm (lower left) and 100 µm (lower right). **(G)** High-resolution stitched images of Ki67 and IBA-1 antibody immunostaining. Bottom image shows a sectional view of the rectangle in the top panel. Scale bars, 100 µm (up) and 20 µm (below). **(H)** Shapes of microglial aggregate. Arrows, microglial orientation. The dashed line shows the IBA-1^+^ cell-enriched site. Scale bar, 200 µm. **(I and J)** Microglia in the border regions of proliferative microglial aggregates were imaged using SIM. The microglia on the border facing the cortical layers (superficial) lack long-oriented processes; the microglia on the border facing the caudate (deeper) show orientation and multiple extended processes (I). Microvascular scaffolding microglia are at the border of the caudate (J). Scale bars, 10 µm. **(K and L)** Illustration of the angle between the vertical line representing a straight line from the meninges to the ventricle and the cellular long-body axis (K). The average angle between the caudate surface facing the body and the vertical axes was 17−20°; the cortical-facing angle ranged from 35° to 41°. The vertical line is the line from the meninges to the ventricles (fetuses, *n* = 5; all data, mean ± SD; Mann–Whitney *U* test, **P < 0.01).

**Figure S2.**
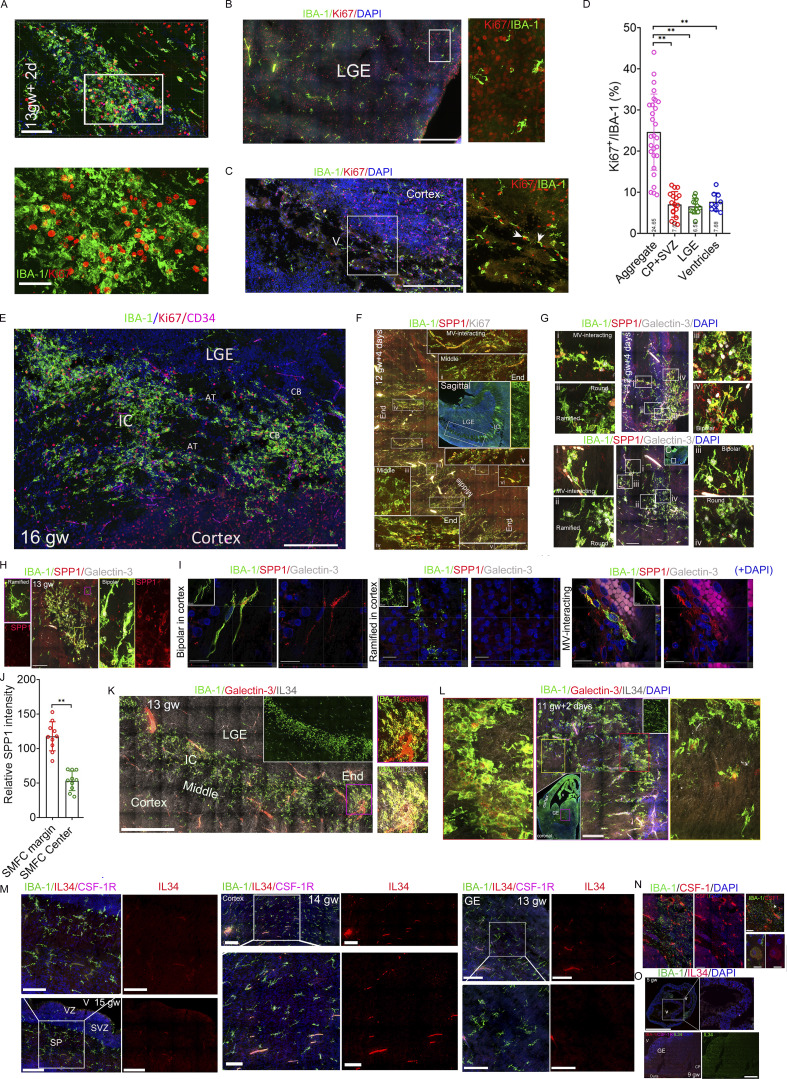
**Anatomical location of the proliferative microglial aggregates and the distribution patterns of SPP1** ^**+**^ **bipolar and MV-interacting microglia in the fetal brain (related to Figs. 2, 3, 4 and 5). (A)** 3D stitching images of IBA-1 and Ki67 antibody immunostaining in a fetal brain (13 gw + 2 days) revealed large microglial aggregates with abundant Ki67^+^ microglia; rectangle, the magnified region in the lower panel (3D displayed as maximum intensity projection [MIP]). Scale bars, 100 µm (top) and 50 µm (bottom). **(B and C)** 3D stitching images of IBA-1 and Ki67 antibody immunostaining in the LGE and the ventricle of fetal brains (15 gw). Boxed, the magnified region in the right (white arrowheads, Ki67^+^/IBA-1^+^ cells). Scale bars, 100 µm (top) and 50 µm (bottom). Scale bars, 500 µm. **(D)** Percentage of Ki67^+^/IBA-1^+^ cells in microglial aggregate, CP, VZ, LGE, and ventricles in 13−16-gw fetal brain (fetuses, *n* = 8; data, mean ± SD; one-way ANOVA with Tukey’s multiple comparisons; **P < 0.01). **(E)** Stitched image of immunostaining with IBA-1 and Ki67 antibodies in the fetal brain at 16 gw. AT, axon tract; CB, cellular bridge. Scale bar, 500 µm. **(F)** SPP1, Ki67, and IBA-1 antibody immunostaining in the sagittal sections of a fetal brain at 12 gw + 4 days. The middle (IBA-1 and DAPI) shows a whole view of the LGE and IC in a sagittal section. The rectangle in the full-view image is the magnified region with a large microglial aggregate (main image). The magnified inserts (i−iv) showed SPP1 immunostaining intensity in the different sections of microglial aggregate (i, MV-interacting microglia with moderate SPP1; ii and iii, aggregate microglia with weak SPP1 in the middle fraction; v and vi, aggregate microglia with strong SPP1 in the ends, vi, a ramified resident microglia with no SPP1 in LGE). The right panel of the whole-view image is the IBA-1 channel, which displays the shape of the microglial aggregate. Scale bar, 1 mm. **(G)** SPP1, Ki67 (top)/galectin-3 (bottom), and IBA-1 antibody immunostaining in the coronal sections of a fetal brain at 12 gw + 4 days. The rectangle in the whole-view image (IBA-1 and DAPI; below) corresponds to the magnified region shown in the inset. The magnified inserts (i−iv) showed SPP1 intensity in different sections of the microglial aggregate. Top: i, MV-interacting microglia with moderate SPP1; ii and iii, ramified resident microglia with no SPP1 (ii) and SMFC microglia with weak SPP1 in the middle (iii); iv, aggregate bipolar microglia with strong SPP1 in the margin. Bottom: i, MV-interacting microglia with moderate SPP1 and galectin-3; ii and iii, aggregate center microglia with weak SPP1 and strong galectin-3; iii, bipolar microglia in the margin with strong SPP1 and strong galectin-3; iv, the aggregate center microglia with no SPP1 and strong galectin-3. Scale bars, 100 µm. **(H)** SPP1, galectin-3, and IBA-1 antibody immunostaining in the coronal section of a fetal brain at 13 gw. The yellow rectangle is the magnified region on the right that shows SPP1^+^ and galectin-3^+^ bipolar microglia, and the magenta rectangle is the magnified region on the left that shows a ramified microglia with weak SPP1 and galectin-3. Scale bars, 200 µm. **(I)** Sectional view of the SPP1, galectin, and IBA-1 immunostaining in a fetal brain at 13 gw shows the SPP1 and galectin expression patterns in scattered bipolar and ramified microglia. Bipolar microglia are SPP1^+^ but do not express galectin-3 (first and last); the ramified microglia with multiple bulbous endings do not express SPP1 and galectin-3 (middle); the MV-interacting microglia express a high level of SPP1 but do not express galectin-3. Scale bars, 20 µm. **(J)** SPP1 expression levels in the marginal and center of microglial aggregate (fetuses, *n* = 4; dot, a region; Mann–Whitney *U* test; **P < 0.01). **(K)** IL-34, galectin-3, and IBA-1 antibody immunostaining in the sagittal (D) sections of a fetal brain at 13 gw showed the main section and one end of a microglial aggregate. The magenta rectangle in the right panel shows a magnified image of the microglia at the end of the aggregate, which highly express galectin and harbor abundant IL-34^+^ cells. Scale bars, 500 µm. **(L)** IL-34, galectin-3, and IBA-1 antibody immunostaining in the coronal sections of a fetal brain at 11 gw + 2 days. The inner insert (lower left) shows the entire coronal section. The magenta rectangle in the whole-view image is the magnified image in the middle. The red rectangle is a magnified region of the left panel; the yellow rectangle in the right panel shows a few IL-34^+^ cells in aggregate. Scale bars, 200 µm. **(M)** Different fetal brain regions with scattered microglia (13−15 gw) immunostained by IL-34, IBA-1, and CSF-1R antibodies. Right, IL-34^+^ signals. Imaging, Olympus SpinSR10 spinning disk confocal super-resolution microscope (stitched and Z-stacked; interval, 1 μm; objective, 60 oil). V, ventricle; SP, subplate. Scale bars, 200 µm (left large views), 100 µm (upper right), and 10 µm. **(N)** Immunostaining of the fetal brain with IL-34, IBA-1, and CSF-1 antibodies revealed that CSF-1 is weakly expressed in most IBA-1^+^ microglia in the SMFC (white arrowhead, magnified cell). Scale bars, 200 µm (left large view), and 10 µm (magnified). **(O)** 5- and 9-gw fetal brain regions stained by IL-34 and IBA-1 antibodies. Boxed, the magnified regions. Scale bars, 400 µm. MV, microvasculature.

**Figure 3.**
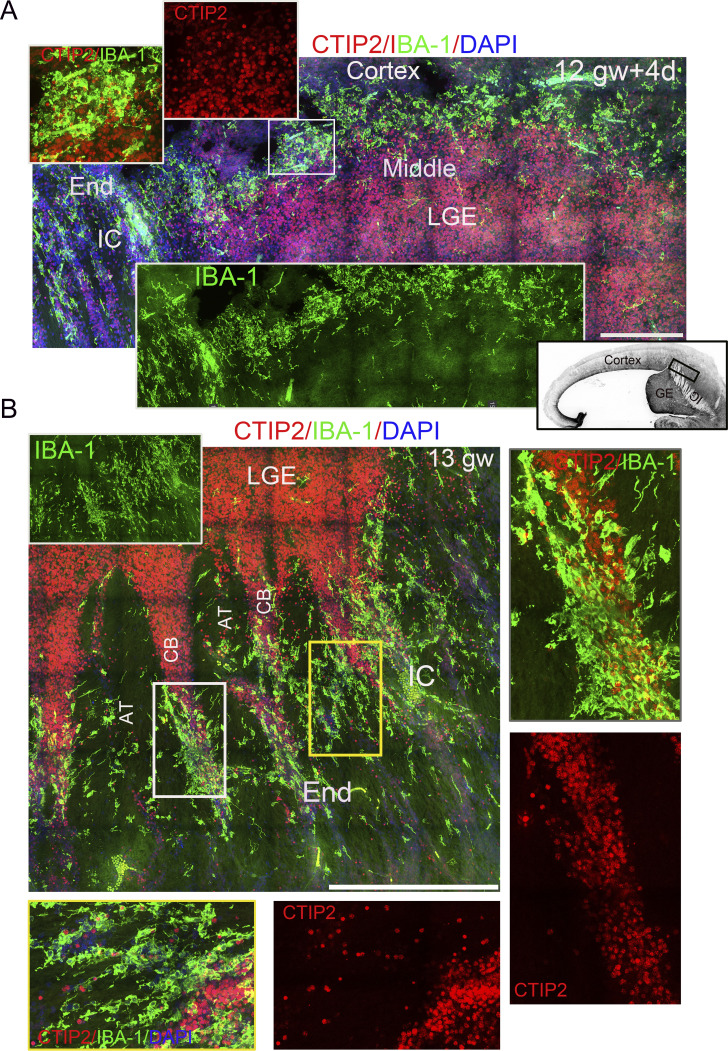
**Locations of large proliferative microglial aggregates in the fetal brains. (A and B)** 3D stitching images of IBA-1 and CTIP2 antibody immunostaining in a fetal brain at 12 gw + 4 days (A) and 13 gw (B) revealed the anatomical position of the microglial aggregate. The upper left insert is a magnified view of the rectangle, showing the position of the middle fraction of the microglial aggregate (A). The black rectangle in the lower right image (black and white image of the whole brain region) indicates the position of the large image in the brain (A). The magnified image on the right, from the white rectangle, shows the relationship between the cellular bridge and the microglial aggregate. The magnified image below (B), from the yellow rectangle, shows that some cellular bridges are composed of microglial aggregates. The green channel insertions (A and B) show the IBA-1 signal: AT, axon tract; CB, cellular bridge. Scale bars, 200 µm (A) and 500 µm (B).

While screening the 50-µm sections, we identified a large and dense Ki67^+^ microglial aggregate near the caudate in the fetal brains at 12.5–16 gw (*n* = 10). All microglial aggregates contain rich Ki67^+^/IBA-1^+^ cells, whereas cortical, lateral ganglionic eminence (LGE), and ventricular regions lack such high densities of Ki67^+^ microglia (Fig. 2, A–D; and Fig. S2, A–E). Ki67^+^ microglia in those aggregates account for 26.65% (4,625/17,352) of all aggregate microglia, and 6.32% (209/3,306) of all scattered microglia in other regions (Table S2). The average Ki67^+^ microglial percentage is around 6.5–7.5% in the SVZ, CP, and ventricle, and 24.65% in microglial aggregates, remaining stable from 13 to 16 gw (Fig. S2 D and Fig. 2 E). By estimating microglial aggregate size using nine consecutive sections from 15-gw fetal brains, the largest aggregate measured 1.736 mm^2^ (total area of nine sections: 6.88 mm^2^) (Fig. 2 F). Observably, Ki67^+^ round microglia are richer and denser in the center of microglial aggregates, whereas ramified or bipolar microglia dominate in the margins (Fig. 2 G). The large proliferative microglial aggregates coincide with the emergence of the oSVZ in human fetal brains, which contributes to the large and complex human brain (Hansen et al., 2010). Extended bipolar morphology is a signature of migrating microglia (Dormann and Weijer, 2006), and the long axis angle of bipolar microglia indicates the orientation (Wei et al., 2009). Notably, the marginal microglia of the aggregate are oriented toward deep brain structures (Fig. 2, H and I). The microvasculature-attaching marginal microglia in aggregate also oriented the deep caudate (Fig. 2 J). The acute angle of the marginal microglia toward the caudate and the superficial marginal zone is 20° and 40°, respectively (Fig. 2, K and L), suggesting directional alignment in the marginal microglia (Lois and Alvarez-Buylla, 1994; Menassa and Gomez-Nicola, 2018). Anatomically, the two ends of the proliferative microglial aggregate in 12 gw + 4 days (12.5)−16 gw fetal brains are located in the CTIP2^+^ cellular bridge of the internal capsule (IC) distal to the LGE, and the middle fraction appeared in the cortical layer next to the CTIP2^+^ LGE (Fig. 3, A and B). Therefore, the proliferative microglial aggregate might be a specific microglial expansion center in the human fetal brain cortex during the oSVZ stage.

### The large proliferative microglial aggregate differs from previously identified microglial clusters despite partial overlap

**Figure 4.**
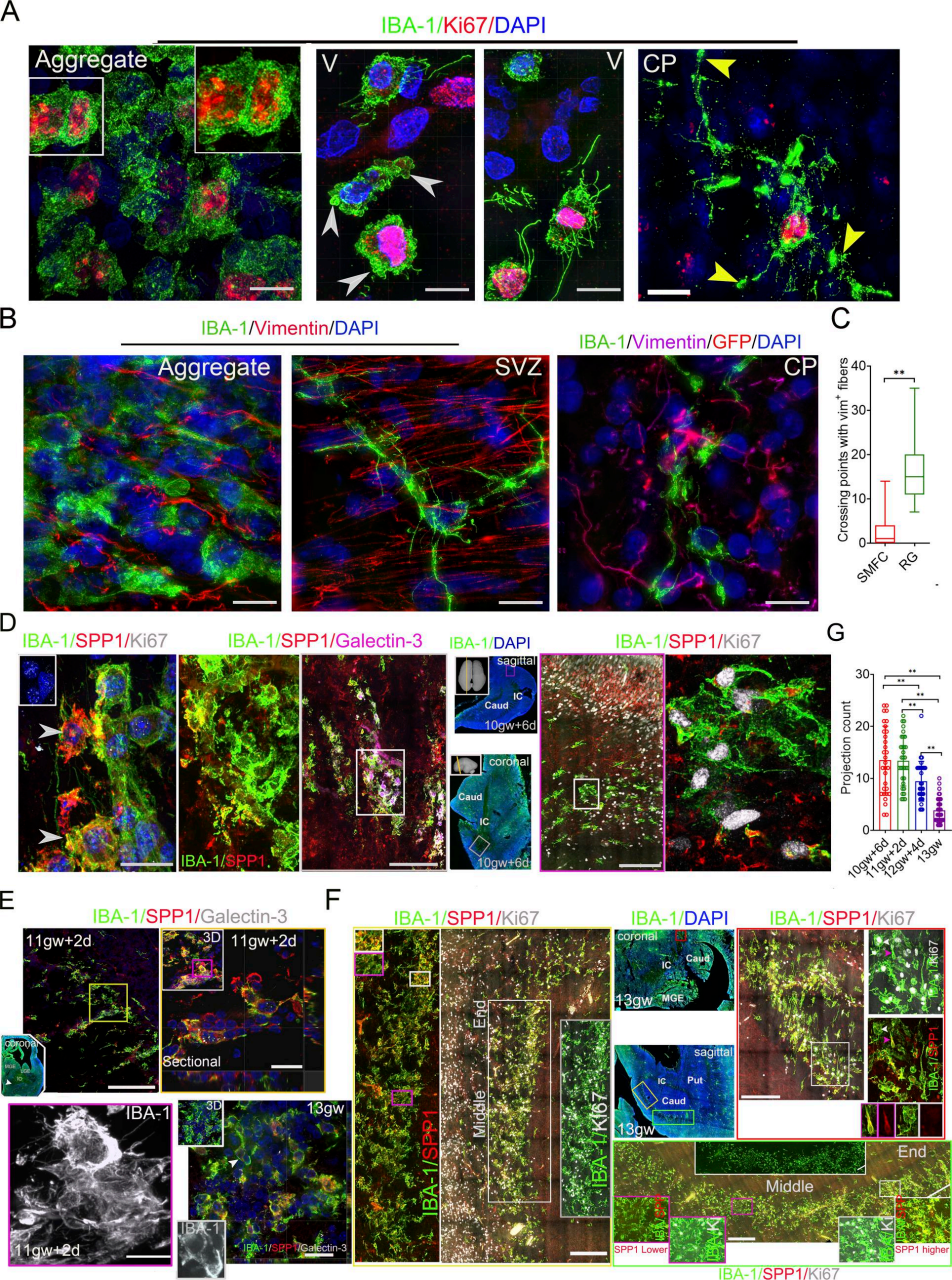
**Microglial aggregates contain a distinct round proliferative microglia in the center and SPP1** ^**+**^ **bipolar and MV-interacting microglia along the border region. (A)** IBA-1 and Ki67 antibody immunostaining in microglial aggregates, V, and CP revealed the distinct morphology of microglia in microglial aggregates. The microglia in the proliferative aggregate are uniformly round (left), including proliferative or nonproliferative; the amoeboid microglia in the ventricle have membrane ruffles, and Ki67^+^ microglia in the ventricle exhibit several slender processes and membrane ruffles (middle). White arrowheads, membrane ruffles; yellow arrowheads, bulbous ending. RG-interacting Ki67^+^ microglia exhibit long projections and bulbous endings (right). Recording, SIM microscopy. Scale bars, 10 µm. **(B)** Immunostaining with vimentin, GFAP (right only), and IBA-1 antibodies in aggregate, SVZ, and CP revealed the relationships of microglial projections with vimentin^+^ or GFAP^+^ fibers. Scale bars, 10 µm. **(C)** Cross-point count in RG scaffolding and aggregate microglia with vimentin^+^ fibers (data, mean ± SD; Mann–Whitney *U* test; **P < 0.01). **(D)** SPP1, galectin-3 or Ki67, and IBA-1 antibody immunostaining in the coronal and sagittal sections of a fetal brain at 10 gw + 6 days. The middle (IBA-1 and DAPI) shows the coronal (top) and sagittal (bottom) sections (inner insert, sectioning position). The rectangles (magenta [coronal], the magnified region in the right with the magenta border; gray [sagittal], the magnified region in the left with the gray border) are a region of microglial aggregate. The super-resolution images on the right revealed that the SPP1^+^ microglia display multiple projections. Scale bars, 20 µm (left [first]), 50 µm (middle [third]), and 100 µm (right [fifth]). **(E)** SPP1, galectin-3, and IBA-1 antibody immunostaining in the coronal sections of a fetal brain at 11 gw + 2 days. The arrow (white) in the whole view of the brain section indicates the magnified region in the upper left panel. The rectangle (yellow) is the magnified region (up, right), which is a sectional view that displays the morphology of the aggregate microglia and SPP1 and galectin-3 staining intensity (strong). The gray-channel image (below) from the rectangle region of the upper right panel shows IBA-1 staining, revealing microglial processes. Scale bars, 200 µm (upper left), 10 µm (upper right), and 10 µm (lower [super-resolution]). **(F)** SPP1, Ki67, and IBA-1 antibody immunostaining in the coronal and sagittal sections of a fetal brain at 13 gw. The middle (IBA-1 and DAPI) shows a whole view of the coronal (top; inner insert, the sectioning position) and sagittal (bottom) sections. The rectangles (red [coronal], the magnified region in the right with the red border; yellow and green [sagittal], the magnified region in the left with the yellow border, and below with a green border) are a region of microglial aggregate. The left (IBA-1 and SPP1) in the yellow rectangle is a magnified image of the rectangle on the right (the inner insert with a white border shows microglia with high SPP1 at the end of the microglial aggregate; the inner insert with a yellow border shows microglia with low SPP1 in the middle section of the long microglial aggregate). The lower right image, with a green border, is a magnified view of the region within the green rectangle in the sagittal section (the inner insert with a magenta border [lower left]), the magnified image from the magenta rectangle, and microglia with low SPP1 in the middle section of a long microglial aggregate. The inner insert with a white border [lower right] shows microglia with high SPP1 at the end of the microglial aggregate. The right panel in the image with a red border is a magnified image from the white rectangle in the left (the white arrow is a Ki67^+^ round cell with no SPP1 [lower image with white border]; the pink arrow is a bipolar cell with high SPP1 [lower image with pink border]). The right [lower] image is a super-resolution image (sectional view) of IBA-1, SPP1, galectin-3 antibody immunostaining (the inner insert [lower], IBA-1–stained microglia; the inner insert [upper], 3D view). Scale bars, 200 µm (left), 50 µm (upper right), 100 µm (lower right), and 20 µm (lower left [super-resolution]). **(G)** Counting the projections of microglia in the microglial clusters/aggregates (10 gw + 6 days, 11 gw + 2 days, 12 gw + 4 days, and 13 gw) in super high-resolution images taken under 100× oil objective (data, mean ± SD; one-way ANOVA with Tukey’s multiple comparisons, **P < 0.01). V, ventricle.

The large proliferative microglial aggregate anatomically overlaps with a reported 3b microglial cluster, which is speculated to be a “waiting site” that removes lavish axons, promotes neuroaxonal growth, and mediates axonal pathways (Verney et al., 2010). The Ki67^+^ or Ki67^−^ round microglia in the aggregate center lacked membrane ruffles of Ki67^+^ or Ki67^−^ ventricular amoeboid microglia and Ki67^+^ microglial long projections with bulbous endings in SIM 3D images (Fig. 4 A). These cells resided within the vimentin^+^ fibers, which lack an orientation, and their short processes rarely interacted with vimentin^+^ fibers compared with RG-interacting or CP microglia (Fig. 4, B and C). A reported SPP1^+^/galectin-3^+^ microglial cluster in 9–14-gw human fetal brains, especially those spanning the IC in 14-gw fetal brains (Lawrence et al., 2024), also partially overlapped with the large proliferative microglial aggregate in coronal sections (>12.5 gw). SPP1, also known as osteopontin, is widely expressed across many cell types, including tumor cells and multiple immune cells (Gu and Muller, 2025), and enhances cell migration (Wu et al., 2022; Yi et al., 2022). We used SPP1 and galectin-3 antibodies to evaluate their expression patterns of SPP1 and galectin-3 in the sagittal and coronal sections of human fetal brains at 10−13 gw. SPP1^+^ microglia in the smaller microglial cluster of fetal brains at 10 gw + 6 days and 11 gw + 2 days (Fig. 4, D and E), or the larger microglial aggregates of fetal brains at 12 gw + 4 days and 13 gw show ramified structure with multiple long processes or bipolar morphology (Fig. S2, F–H; and Fig. 4 F). SPP1^+^ microglia are mainly distributed in the margin or the two ends of the larger microglial aggregate (>12.5 gw) (Fig. S2, F and G; and Fig. 4 F). Strikingly, the round microglia, including Ki67^+^ and Ki67^−^ microglia, within the larger microglial aggregate (>12.5 gw) rarely express SPP1 (Fig. S2, F and G; and Fig. 4 F). Microglia in microglial clusters (<12 gw) exhibit more complex processes than those in the SMFC (Fig. 4 G). In the cortical layer or the striatum, long bipolar microglia and microvasculature-interacting bipolar microglia highly expressed SPP1, whereas cortical ramified resident microglia with bulbous endings did not express SPP1 (Fig. S2 I). The SPP1 expression levels in the margin of microglial aggregate (>12.5 gw) are higher than those in the center (Fig. S2 J). Most microglia, including round, bipolar, and ramified, in the smaller microglial clusters (<12 gw) or the larger microglial clusters (>12.5 gw) expressed galectin-3, with particularly strong expression in the microglial cluster r (<12 gw) (Fig. 4, D, E, and G; and Fig. S2, G, H, K, and L). In the scattered microglia of fetal brains, galectin-3 was not present in resident ramified microglia with multiple bulbous endings, SPP1^+^ long bipolar microglia, or some SPP1^+^ microvasculature-interacting microglia (Fig. S2, G, H, and I). The large proliferative microglial aggregates in fetal brains (>12.5 gw) are a novel structure with distinct cellular morphology, anatomical location, and molecular signatures, and differ from previously reported microglial clusters. Here, we refer to it as the SMFC.

### SMFC regions increase CSF-1R levels and enrich IL-34^+^ cells

**Figure 5.**
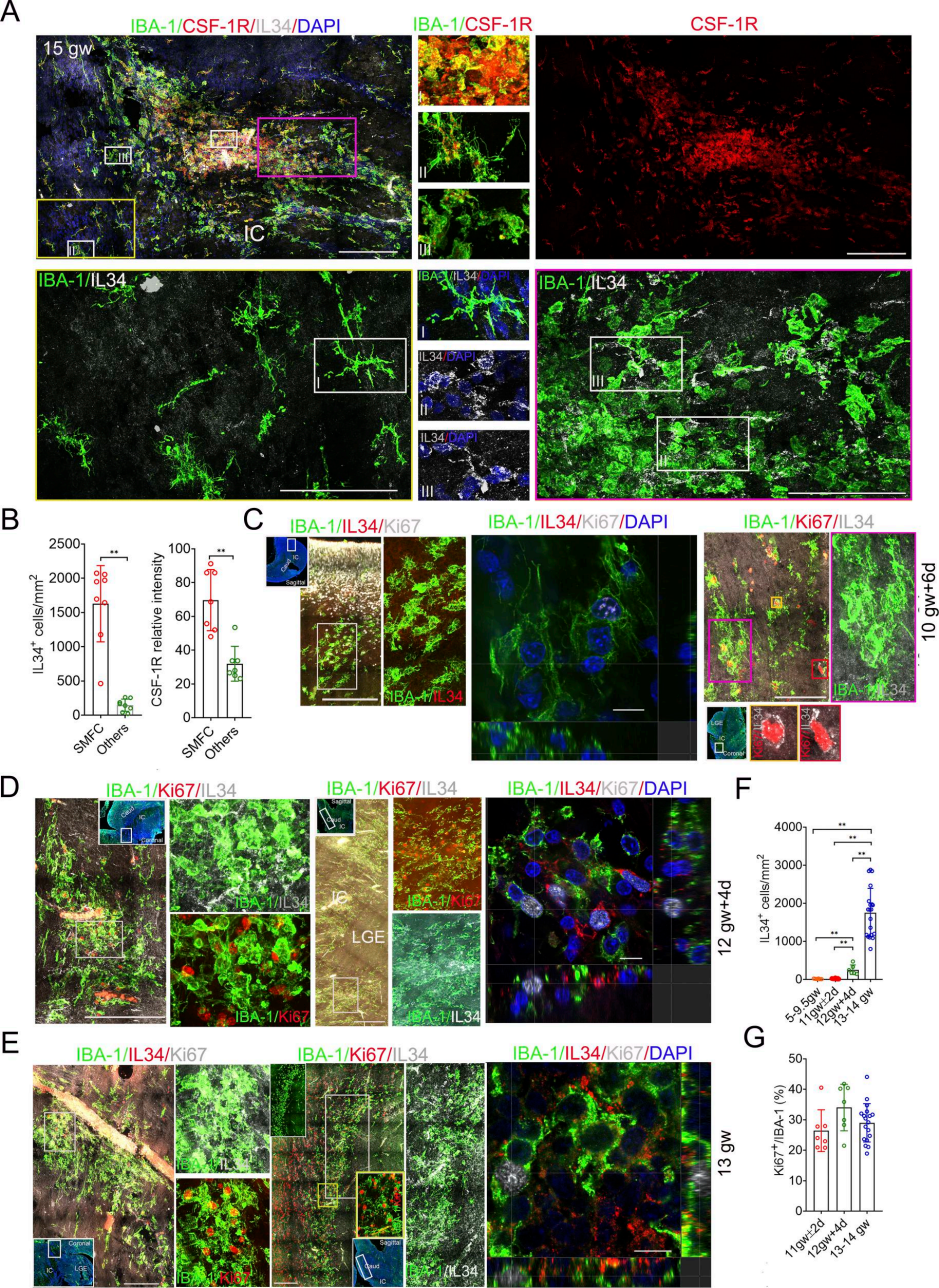
**IL-34** ^**+**^ **cells and high CSF-1R microglia accumulate in the SMFC of the fetal brain. (A)** Representative stitched images of IBA-1, CFS-1R, and IL-34 antibody immunostaining revealed that the microglia in the SMFC express higher levels of CFS-1R and harbor many IL-34^+^/IBA-1^−^ cells (boxed region with yellow or magenta border, the magnified region with yellow or magenta border [below]; the smaller rectangles [top: I, II, III], the magnified region in the middle [CSF-1R and IBA-1]: I, microglia with high CSF-1R in the center of the SMFC; II, scattered microglia with lower CSF-1R; III, microglia with lower CSF-1R in the margin of the SMFC; the rectangles [bottom: I, II, III], the magnified region in the middle [IL-34, IBA-1]: I, scattered microglia region without IL-34^+^ cells; II, III, SMFC region with rich IL-34^+^ cells). Scale bars, 200 µm (top) and 100 µm (bottom). **(B)** IL-34^+^ cell density in the SMFC and other regions (left); relative CSF-1R levels in the SMFC and the scattered microglia of other regions (right) (fetuses, *n* = 7; Mann–Whitney *U* test; **P < 0.01). **(C)** IBA-1, IL--34, and Ki67 antibody immunostaining in the coronal and sagittal sections of a fetal brain at 10 gw + 6 days. The left two panels show the magnified image of the white rectangle in the full-view image (inner insert in the upper left, sagittal). The magnified image of the second panel (left) from the first panel on the left reveals that no IL-34^+^ cells are present in the region of the IBA-1 microglial aggregate. The super-resolution images in the middle revealed that Ki67^+^ aggregate microglia display multiple projections (sectional view). The right two panels from the magnified images of the rectangle in the whole-view image (lower [first], coronal) display microglial clusters, and sporadic IL-34^+^/Ki67^+^ cells in microglial aggregates (the lower two cells, typical IL-34^+^/Ki67^+^ cells). The right panel, with a pink border, is a magnified region of the pink rectangle, showing that IL-34^+^ cells are rare. Scale bars, 200 µm (left), 10 µm (middle), and 100 µm (right). **(D and E)** IBA-1, IL-34, and Ki67 antibody immunostaining in the coronal and sagittal sections of two fetal brains at 12 gw + 4 days and 13 gw. The left two panels show a magnified view of the white rectangle in the whole-view image (inner insert in upper right, coronal). The magnified image of the second panel (left) from the first panel on the left reveals that many IL-34^+^ cells are present in the SMFC region (E and F). Some of the IL-34^+^ cells are Ki67^+^ (E). The middle two panels show magnified images of the rectangle in the whole-view image (sagittal), revealing abundant IL-34^+^/Ki67^+^ cells in the SMFC. The super-super-resolution images (sectional view) on the right revealed that Ki67^+^ cells in the SMFC are small and round (E and F). Scale bars (D), 100 µm (left), 200 µm (middle), and 10 µm (right); scale bars (E), 200 µm (left), 400 µm (middle), and 10 µm (right). **(F)** IL-34^+^ cell density in the SMFC (12.5 gw and 13−14 gw) and microglial cluster (11 gw ± 2 days) and other brain regions (5−9.5 gw) (fetuses, *n* = 13; one-way ANOVA with Tukey’s multiple comparisons; **P < 0.01). **(G)** Percentage of Ki67^+^ in microglial cluster (<12 gw) and the SMFC (>12.5 gw) (fetuses, *n* = 8; one-way ANOVA with Tukey’s multiple comparisons).

The CSF-1R pathway, mediated by CSF-1 and IL-34, controls microglial proliferation and survival in the fetal brain (Elmore et al., 2014). To evaluate the CSF-1R pathway status in the SMFC, we immunostained 5−16-gw fetal brain sections, including coronal and sagittal sections (50 µm), with IL-34, IBA-1, CSF-1R, or CSF-1 antibodies. Notably, the SMFCs harbor rich IL-34^+^/IBA1^−^ cells, and their density in the SMFC region is much higher than that in other brain regions, including VZ, SVZ, subplate, CP, and ganglionic eminence (GE) (Fig. 5, A and B; and Fig. S2 M). CSF-1 is weakly expressed in the microglia of the SMFC, and a few CSF-1^+^/IBA-1^+^ cells are present in the margin of the SMFC (Fig. S2 N). SMFC microglia, especially in the center, expressed higher levels of CSF-1R compared with the scattered microglia in other brain regions (Fig. 5, A and B). The microglial clusters in the coronal and sagittal sections of fetal brains at 10 gw + 6 days or 11 gw + 2 days harbor sporadic IL-34^+^ cells, some of which express Ki67 (Fig. 5 C and Fig. S2 L). In the 12 gw + 4 day fetal brain, the SMFCs harbor a moderate density of IL-34^+^/IBA-1^−^ cells compared with the high IL-34 density in the SMFCs of fetal brains at 13−14 gw, and 13% (113/875) of IL-34^+^/IBA-1^−^ cells are Ki67^+^ (Fig. 5, A and D–F; and Fig. S2 I). IL-34^+^/IBA1^−^ cells are also rare in the cortical and GE regions of 5–9.5-gw fetal brain (Fig. S2 O). Significantly, the percentage of Ki67^+^ microglia in the SMFC with a high IL-34^+^ cell density (>12.5 gw) is identical to that in microglial clusters with few IL-34^+^ cells (<12 gw) (Fig. 5 G). These findings establish that high-density IL-34–expressing cells constitute another distinct signature of the SMFC, suggesting that the IL-34-CSF-1R pathway may regulate the SMFC formation.

### The IL-34-CSF-1R pathway drives the SMFC formation in chimeric microglia–brain organoids

**Figure 6.**
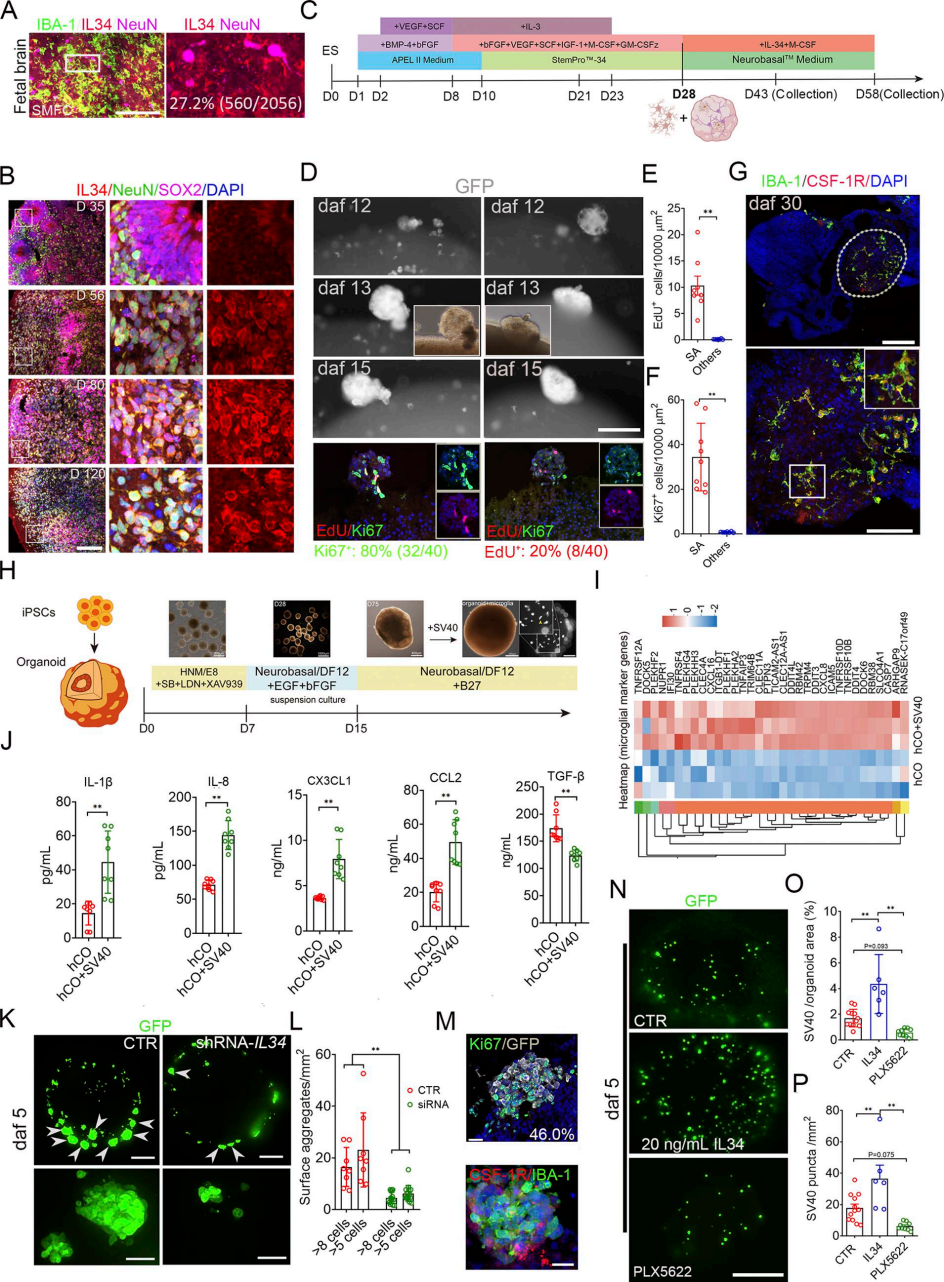
**Recapitulation of the SMFC in chimeric microglia–human hCOs. (A)** NeuN, IBA-1, and IL-34 immunostaining in the SMFC of a 15-gw fetal brain demonstrating that IL-34^+^ cells are NeuN^+^. Scale bar, 100 µm. **(B)** NeuN, SOX2, and IL-34 antibody immunostaining in hCOs at 35 (first row), 56 (second row), 80 (third row), and 120 (fourth row) days, indicating uniform IL-34 expression in hCO neurons. Scale bar, 200 µm. The rectangle, the magnified region in the middle and right. **(C)** Schematic of iMicroglia (iMacrophage) induction from ESC/hiPSCs and the timeline for generating chimeric iMicroglia-hCOs. **(D)** Longitudinal tracking of the surface microglial aggregate after introducing iMicroglia derived from ES-GFP from daf 12 to daf 15. Bottom, EdU labeling (red) and Ki67 immunostaining (green) in the iMicroglia-hCOs of the upper panel revealed that the surface aggregates harbor a high percentage of proliferative cells (the inner insert in the second panel; phase-contrast image of surface aggregates; inner insert in the lower, spliced Ki67^+^ and EdU^+^ signals). Scale bars, 200 µm. **(E and F)** Quantification of EdU^+^/Ki67^+^ cells in iMicroglia aggregates versus nonaggregate regions of hCOs (hCOs, *n* = 6; data, mean ± SD; Mann–Whitney test *U* test; **P <0.01). **(G)** Distribution patterns of iMicroglia in iMicroglia-hCOs stained by IBA-1 and CSF-1R antibodies. The white dashed line circled the necrotic regions. Below is the magnification of the necrotic region (inner insert, a highly ramified microglia in the rectangle). Scale bars, 200 µm (top) and 100 µm (bottom). **(H)** Schematic detailing the integration of SV40-immortalized microglia into hCOs. Microglia are introduced on day 30 by suspending the medium with SV40 microglia. **(I)** Heatmap displaying upregulated genes encoding microglia-related cytokines and proteins in SV40-hCOs. **(J)** Concentrations of IL-1β, IL-8, TGF-β, CCL2, and CX3CL1 in the supernatants of SV40-hCOs versus control hCOs (data, mean ± SD; Mann–Whitney *U* test; **P < 0.01). **(K)** Representative whole-view images of SV40 microglia in SV40-hCOs (left) and hCOs subjected to IL-34 knockdown via shRNA lentivirus particles (right) (below, the magnified surface aggregate; white arrowheads, surface aggregate). Recording, the long-distance object of Olympus SpinSR10 spinning disk confocal microscopy with Z-stack (intervals, 1 µm). Control, CTR. Scale bars, 200 µm (top) and 50 µm (bottom). **(L)** Quantification of microglial aggregates (>5 or >8 cells) in control versus IL-34-shRNA SV40-hCOs (*n* > 3 repeats; hCOs: CTR, *n* = 8; shRNA, *n* = 15; Mann–Whitney *U* test; **P < 0.01). **(M)** Immunostaining with Ki67/CSF-1R and GFP antibodies in SV40-hCOs shows that most microglia in the surface aggregate expressed Ki67 (top) and CSF-1R (bottom). Scale bars, 20 µm. **(N)** Reconstructed whole-view images of SV40 microglia in SV40-hCOs treated with PLX5622 (administered before introduction) and 20 ng/ml IL-34 (administered during coculture). Recording, 20× long objective of the Olympus SpinSR10 spinning disk confocal super-resolution microscope with 4-μm intervals in Z-stacks. Scale bar, 500 µm. **(O and P)** Quantification of aggregate size and count in treated SV40-hCOs (data are presented as the mean ± SD; one-way ANOVA with Tukey’s multiple comparisons; **P < 0.01).

**Figure S3.**
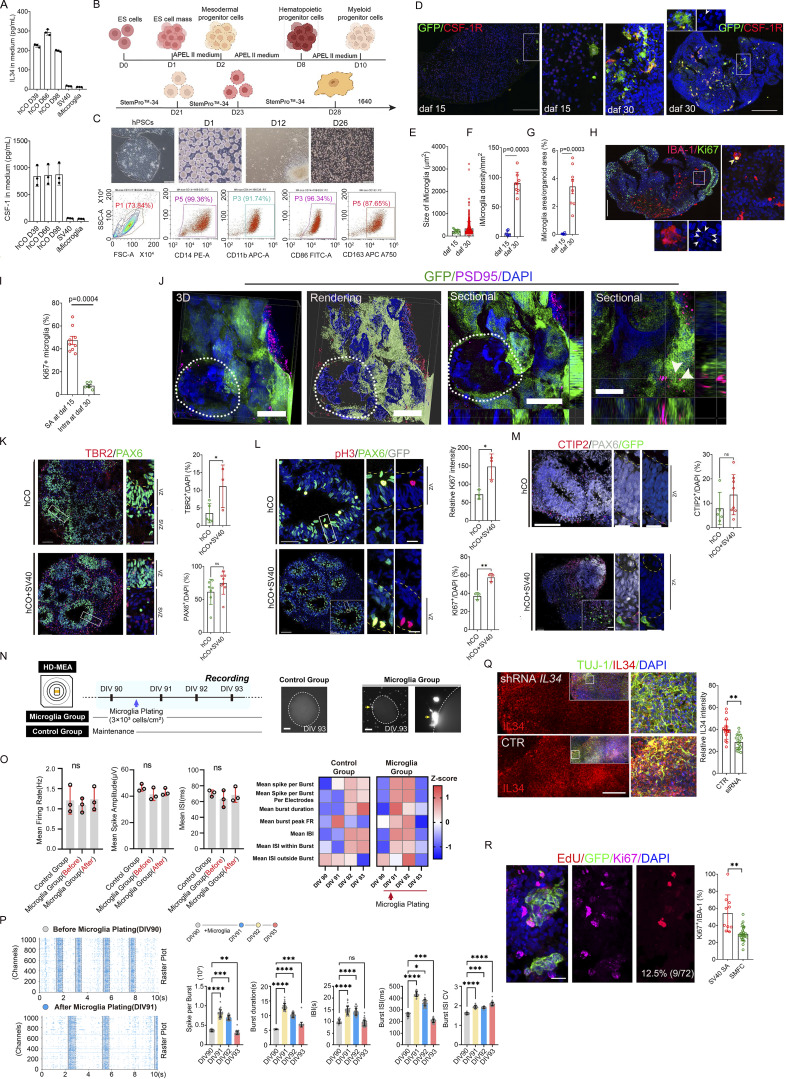
**Characterization of hCOs and chimeric iMicroglia-hCOs (related to Fig. 6). (A)** Evaluating endogenous IL-34 and CSF-1 secretion levels in hCOs and SV40 microglia and iMicroglia. **(B)** Schematics of inducing iMicroglia (iMacrophage) from ESC/hiPSCs and the timeline/strategies for generating and validating iMicroglia-hCOs (B). **(C)** Morphology of iMicroglia during induction and testing the purity of iMicroglia using flow cytometry with CD14, CD11b, CD86, and CD163 antibodies revealed the high purity of iMicroglia. **(D)** Immunostaining of daf 15 and 30 iMicroglia-hCOs with GFP and CSF-1R antibodies revealed the amoeboid morphology of iMicroglia in daf 15 (left two panels) and the ramified morphology of iMicroglia in daf 30 iMicroglia-hCOs (right two panels). Boxed, the middle magnified images (white arrowheads in the upper left, nuclear debris). Scale bars, 200 µm. **(E and F)** Size and density of iMicroglia in daf 15 and daf 30 iMicroglia-hCOs (hCOs, *n* = 4; data, mean ± SD; Mann–Whitney *U* test). **(G)** iMicroglia and iMicroglia area/total region in daf 15 and daf 30 iMicroglia-hCOs (hCOs, *n* > 7; repeat, *n* = 3; data, mean ± SD; Mann–Whitney *U* test). **(H)** Whole slide scanning of daf 30 iMicroglia-hCOs immunostained with Ki67 and IBA-1 antibodies revealed that a restricted number of intra-hCO iMicroglia express Ki67, and the iMicroglia included multiple DAPI-stained nuclear fragments. Right, the boxed region (yellow arrow, Ki67^+^ iMicroglia; white arrowheads in the lower panel, nuclear debris). Scale bars, 200 µm. **(I)** Percentage of Ki67^+^ microglia in daf 30 iMicroglia-hCOs (intra-organoid) and Ki67^+^ surface iMicroglia in daf 15 iMicroglia-hCOs (repeat, *n* = 3; total iMicroglia-hCOs, *n* = 8; Mann–Whitney *U* test; data, mean ± SD). **(J)** SV40 microglia in the hCO stained by GFP, PSD95, and MAP2 antibodies revealed PSD95^+^ and MAP2^+^ puncta in penetrated SV40 microglia (white dashed line, phagocytosed nuclear debris; white arrowheads, PSD95^+^ dots). Recording, SIM. Rendering, IMARIS software. Scale bars, 10 µm. **(K)** Immunostaining for SV40 microglia-hCO or hCO using PAX6 and TBR2 antibodies revealed that more TBR2^+^ cells are present in the SVZ region of hCO with SV40 microglia. Boxed (left), the magnified region (right). Percentage of TBR2^+^ or PAX2^+^ cells in SV40 microglia-hCO and hCO (organoid, *n* ≥ 3; all data, means ± SD; Mann–Whitney *U* test, *P < 0.05). Scale bars, 100 µm (left) and 20 µm (right). **(L)** Immunostaining with Ki67, pH3, and GFP antibodies in hCO with SV40 microglia or hCO. Percentage of Ki67 intensity of Ki67^+^ cells in hCO with SV40 microglia with hCO (organoids: *n* = 3; data, mean ± SD; Mann–Whitney *U* test. *P < 0.05; **P < 0.001). Scale bars, 100 µm (left) and 20 µm (right). **(M)** Immunostaining with CTIP2, GFP, and PAX6 antibodies in SV40 microglia-hCO and hCO. Percentage of CTIP2^+^ cells in SV40 microglia-hCO and hCO. Organoid, *n* ≥3; all data, means ± SD; Mann–Whitney *U* test, ns, P > 0.05. Scale bars, 100 µm (left) and 20 µm (right). **(N)** Schematic illustrates the generation of SV40-hCO chimeras in HD-MEA (left). Living imaging of SV40-hCO chimeras revealed the interaction of GFP + SV40 microglia with hCO. SV40-hCO chimeras were stripped from HD-MEA at the end of recordings (right). **(O)** Quantification of the mean firing rate [Hz], the mean spike amplitude [µV], and the mean ISI [ms] in hCO. *n* = 3 control group or microglia group; The mean ± SD; Kruskal–Wallis test; ns, no significant difference (left). Quantification of network features indicated SV40 microglia-guided remodeling of the neuronal network. The network features include mean spikes per burst, mean spikes per burst per electrode, mean burst duration [s], mean burst peak firing rate [Hz], mean IBI [s], mean ISI within burst [ms], and mean ISI outside burst [ms]. *n* = 3 control group or microglia group (right). **(P)** Raster plots show the dynamic neuronal network in chimeras after SV40 microglial plating (left). The quantification of network features at the burst level in the representative SV40-hCO chimera, including the number of spikes per burst, the duration of the burst [s], the peak firing rate [Hz] of the burst, the IBIs [s], ISI within burst [ms], and ISI outside burst [ms]. The mean ± SD; Kruskal–Wallis test; and statistical significance levels (ns, no significance; *P < 0.05; **P < 0.01; ***P < 0.001; ****P < 0.0001) are presented (right). **(Q)** IL-34 and TUJ-1 antibody immunostaining of the hCOs infected by shRNA and hCO. The inner insert on the left (top) is a multichannel image. The rectangle in the inner insert is the magnified region on the right (left). The relative IL-34 intensity in the hCOs infected by shRNA lentivirus particles and control hCOs (Mann–Whitney *U* test; **P < 0.001) (right). Scale bars, 200 µm. **(R)** Immunostaining with the Ki67 antibody and EdU labeling of the surface aggregate (SA) in SV40-hCOs (left). Comparing Ki67^+^ surface aggregates with Ki67^+^ cells in the SMFC; Mann–Whitney *U* test; **P < 0.001 (right). Scale bars, 20 µm. IBI, interburst intervals.

We found that 27.2% (560/2,056) of IL-34^+^/IBA-1^−^ cells in the SMFC expressed NeuN, a mature neuronal marker (Fig. 6 A). Strikingly, massive IL-34^+^/NeuN^+^ cells were also present in our hCOs (Liu et al., 2024), which secreted IL-34 and CSF-1 (Fig. 6 B and Fig. S3 A). To determine whether integrating microglia into IL-34–expressing hCO replicates the SMFC, we induced H9-GFP, a human embryonic stem cell expressing GFP, into iMicroglia, and introduced the iMicroglia into hCO at day 28 (Fig. 6 C; and Fig. S3, B and C). Longitudinal tracking revealed that a prominent microglial aggregate appeared on the surface of hCO at 12 days after fusion (daf 12) and lasted to daf 15 (Fig. 6 D). At daf 15, 80% (32/40) and 20% (8/40) of the iMicroglia in aggregates are Ki67^+^ and EdU^+^, respectively (Fig. 6 D). The density of EdU^+^ and Ki67^+^ cells in the surface microglial aggregates is higher than that in other regions (Fig. 6, E and F). A limited number of microglia with amoeboid morphology and small processes are inside iMicroglia-hCO at daf 15 (Fig. S3 D). At daf 30, abundant scattered iMicroglia with long processes, large body size, phagocytic nuclear debris, and lower proliferative signatures appeared inside hCOs and were preferentially enriched in the necrotic region of hCOs (Fig. 6 G and Fig. S3, D–I), indicating that the microglial aggregate emerges before the appearance of phagocytic ramified microglia in chimeric iMicroglia-hCOs.

The coculturing medium of chimeric iMicroglia-hCOs contains 20 ng/ml IL-34 and 100 ng/ml CSF-1. Based on the secretion of CSF-1 and IL-34 by hCOs, we constructed another chimeric SV40-immortalized microglia-hCOs (SV40-hCO) without IL-34 and CSF-1 supplements (Fig. 6 H). Like iMicroglia in hCOs, intra-organoids SV40 microglia phagocytosed nuclear fragments or MAP2^+^ fragments/PSD95^+^ puncta in SV40-hCO (Fig. S3 J). SV40-hCOs exhibited upregulated expression of genes related to synaptic and dendrite development, including *BNIP3L*, *MAP2K1*, *PGK1*, *ENO1*, and *IGFBP2* genes, and microglia-specific genes (e.g., *CXCL16*, *NUPR1*, *TNFAIP3*, *TNFRSF19*, *CLEC11A*, and *CLEC4A*), compared with hCOs (Fig. 6 I). SV40-hCOs also showed increased IL-1β, IL-8, CCL2, and CX3CL1 secretion and reduced TGF-β secretion (Fig. 6 J). Compared with hCOs, SV40-hCOs increased TBR2^+^ cells in the SVZ, and Ki67^+^ progenitor cells in the neural tubes, but not PAX6^+^ or CTIP2^+^ neuronal progenitors (Fig. S3, K–M). Electrophysiological profiling via longitudinal high-density microelectrode arrays (HD-MEAs) in SV40-hCOs showed that the mean firing rate, spike amplitude, and interspike intervals (ISIs) were unchanged (Fig. S3, N and P). However, at the network level, network dynamics was significantly remodeled in SV40-hCOs, exhibiting biphasic evolution of network burst strength and synchronization: a transient amplification peaking at 48 h after integration, followed by attenuation at 72 h, in contrast to the progressive intensification observed in control groups (Fig. S3 P, left). Moreover, significant shifts in interburst intervals and intraburst ISI variability underscore a reorganization of network-level bursting dynamics in chimeric models (Fig. S3 P, right). Together, SV40 microglia drive the secretion of proinflammatory cytokines, enhance neuronal maturation, and selectively reconfigure network synchronization in SV40-hCOs, indicating that SV40 microglia are suitable for studying microglial behaviors.

We noticed that microglia also formed multiple aggregates on the SV40-hCO surface on daf 5, and that inhibiting IL-34 expression in the hCO with shRNA (lentiviral particles) reduced the number of surface microglial aggregates (Fig. 6, K and L; and Fig. S3 Q). 46.0% (192/417) of surface aggregate SV40 microglia, which expressed CSF-1R, were Ki67^+^, and 12.5% (9/72) of surface SV40 microglia in the aggregate were labeled by EdU (Fig. 6 M and Fig. S3 R). Furthermore, exogenous administration of 20 ng/ml IL-34 to SV40-hCO increased the size and density of SV40 microglial aggregates, and inhibiting CSF-1R activity with PLX5622 (pretreated), a classical CSF-1R inhibitor (Wen et al., 2023), decreased their size and density (Fig. 6, N–P). Taken together, the IL-34-CSF-1R pathway regulates the SMFC formation in hCOs.

### IL-34 and CSF-1 differentially reshape microglial morphology and transcriptomic profiles in vitro

**Figure 7.**
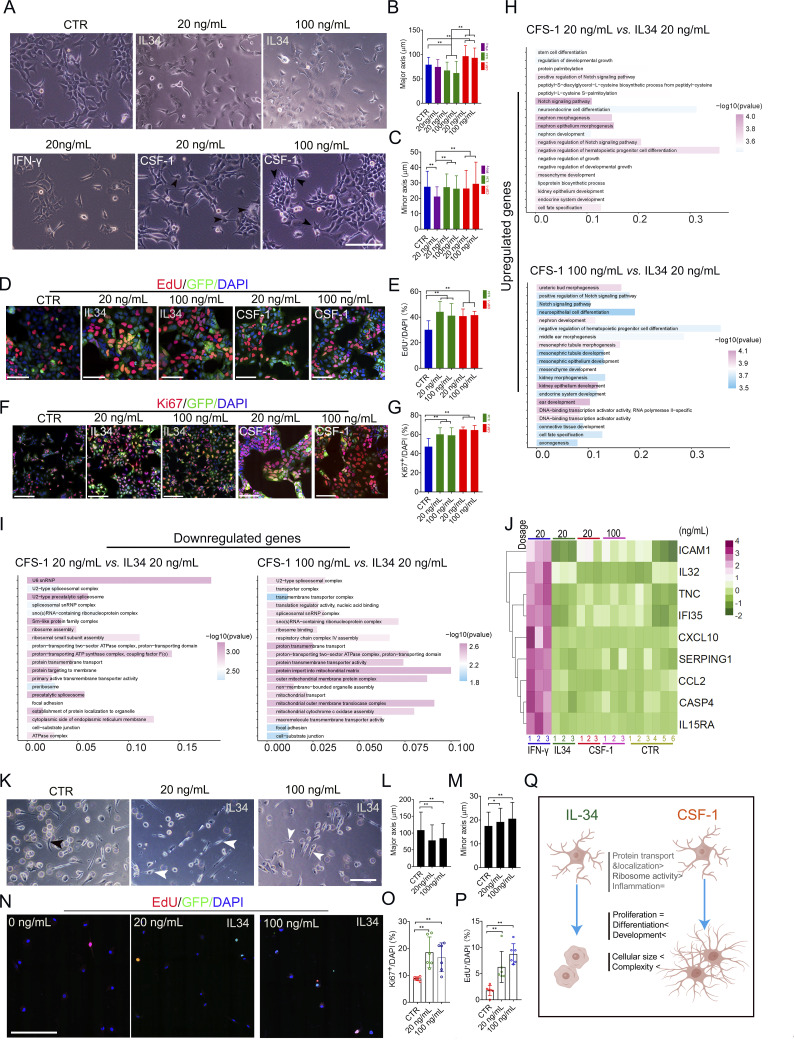
**IL-34 and CSF-1 differentially reshape microglial morphology and transcriptomic profiles in vitro. (A)** Representative morphology of SV40 microglia treated with IL-34 (20 and 100 ng/ml), CSF-1 (20 and 100 ng/ml), and IFN-γ (20 ng/ml) for 120 h (black arrowheads, SV40 microglia with multiple projections). Scale bars, 200 µm. **(B and C)** Quantification of major (B) and minor (C) axis lengths in treated SV40 microglia (*n* = 6 repeats; data are presented as the mean ± SD; one-way ANOVA with Tukey’s multiple comparisons; *P <0.05, **P < 0.01). **(D–G)** EdU labeling (D and E) and Ki67 immunostaining (F and G) of treated SV40 microglia, along with corresponding quantification (*n* ≥4 repeats; data, mean ± SD; one-way ANOVA with Tukey’s multiple comparisons; **P < 0.01). Scale bars, 100 µm (D) and 200 µm (F). **(H and I)** Gene enrichment analysis and functional annotation for upregulated (H) and downregulated (I) genes in SV40 microglia treated by CSF-1 (20 ng/ml and 100 ng/ml) and IL-34 (20 ng/ml). **(J)** Heatmap of bulk RNA-seq data from the SV40 microglia treated with IFN-γ (20 ng/ml), IL-34 (20 ng/ml), CSF-1 (20 and 100 ng/ml), and CTR. **(K–M)** Representative morphology (K) and axis quantification of the major (L) and minor (M) axes of iMicroglia treated with IL-34 (*n* = 4 repeats; data, mean ± SD; one-way ANOVA with Tukey’s multiple comparisons; **P < 0.01). Scale bars, 100 µm. **(N–P)** Ki67 and EdU labeling (N) and quantification (O and P) in IL-34–treated iMicroglia (data, mean ± SD; one-way ANOVA with Tukey’s multiple comparisons; *P < 0.05, **P < 0.01). Scale bar, 200 µm. **(Q)** Schematic comparing the distinct phenotypic effects of IL-34 and CSF-1 on microglia.

To determine whether IL-34 drives the small, round microglia in the SMFC, we tested its effects on microglia, using CSF-1 and IFN-γ as controls. 20 ng/ml and 100 ng/ml IL-34 treatment produced round cells with fewer processes and significantly reduced the major axis in SV40 microglia, whereas 20 and 100 ng/ml CSF-1 produced complex microglia with multiple processes and increased the major axis without affecting the minor axis (Fig. 7, A–C). Compared with CSF-1 and IL-34, 20 ng/ml IFN-γ, a proinflammatory cytokine (Ji et al., 2021), reduced the minor axis of SV40 microglia without affecting the major axis (Fig. 7, A–C). Both 20 and 100 ng/ml IL-34 or CSF-1 treatments increased the number of Ki67^+^ and EdU^+^ SV40 microglia (Fig. 7, D–G). RNA sequencing (RNA-seq) data reveal that both 20 ng/ml and 100 ng/ml CFS-1 primarily increased the transcription of differentiation-, developmental-, and morphogenesis-related genes compared with IL-34 treatment in SV40 microglia (Fig. 7 H). However, IL-34 treatment increases the transcripts of genes involved in protein trafficking/transport, mitochondrial function, ribosome function, and RNA processing compared with CSF-1 treatment (Fig. 7 I). IFN-γ treatment, but not IL-34 and CSF-1 treatment, increased immune response-related genes, including *ICAM1*, *SERPING1*, *IFI35*, *CCL2*, *CXCL10*, *IL15RA*, and *IL32* (Fig. 7 J). Consistent with findings in SV40 microglia, IL-34 treatment increased Ki67^+^ and EdU^+^ cells and reduced the major axis of iMicroglia (Fig. 7, K–P). Those in vitro findings substantiate the hypothesis that IL-34, but not CSF-1, may be the major driver of the SMFC in the human fetal brain (Fig. 7 Q).

### SMFCs are expanded in the brains of fetuses with impaired development

**Figure 8.**
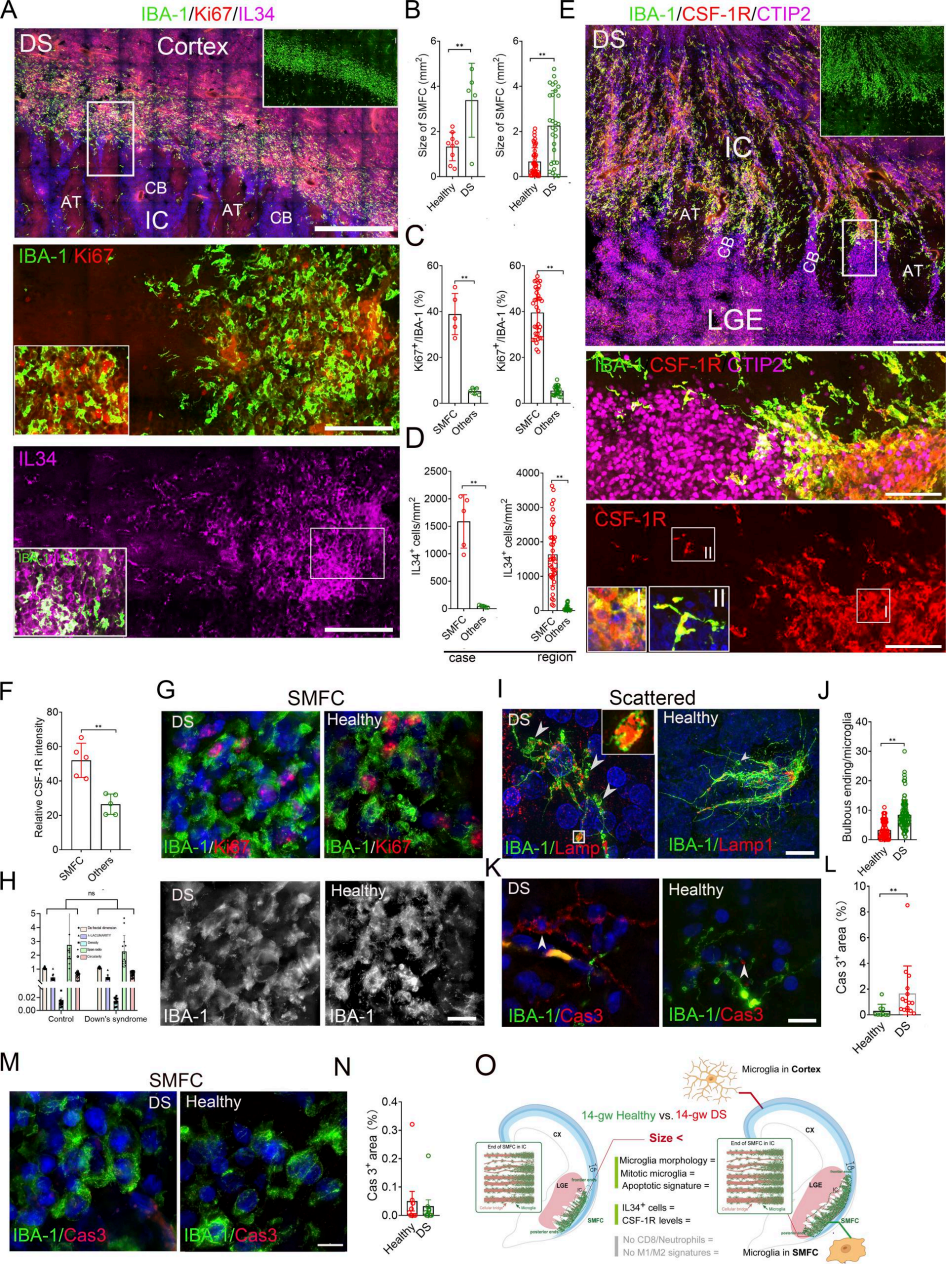
**DS fetal brains harbor an expanded SMFC. (A)**. Stitched high-resolution images of IBA-1, Ki67, and IL-34 antibody immunostaining in a DS fetal brain at 14 gw reveal the main middle section of SMFCs. The inner panel (top right) shows IBA-1^+^ cell density in the SMFC and other regions. The middle panel shows Ki67^+^, IBA-1^+^ cells in the magnification of the rectangle (up; the inner insert, typical high Ki67^+^/IBA-1^+^ density area). The lower panel shows IL-34 signal (top; the inner insert, typical high IL-34^+^/IBA-1^+^ density area). CB, cellular bridge; AT, axon tract. Scale bars, 500 µm (top panel) and 50 µm (middle and lower panels). **(B and C)** Quantification of aggregate size (B) and Ki67^+^ microglial percentage (C) in DS versus healthy fetal brains (DS, *n* = 5; healthy, *n* = 8; data, mean ± SD; Mann–Whitney *U* test; *P <0.05, **P <0.01). Left, by case; right, by region. **(D)** IL-34^+^ cell density in the SMFC of DS and healthy fetal brains (DS, *n* = 5; healthy, *n* = 7; data, mean ± SD; Mann–Whitney *U* test; **P < 0.01). Left, by case; right, by region. **(E)** Stitched images of IBA-1, CSF-1R, and CTIP2 antibody immunostaining in a DS fetal brain at 14 gw reveal that the end of the SMFC extended into the IC. Middle, the magnification of the rectangle (top, without DAPI). Bottom, the magnification of the rectangle (top, CSF-1R only; the inner inserts: SMFC microglia [I] and scattered microglia [II]). Scale bars, 200 µm (top) and 50 µm (middle and bottom). **(F)** CSF-1R staining intensity in the SMFC versus scattered microglia in DS fetal brains (DS, *n* = 5; healthy, *n* = 7; data are presented as the mean ± SD; Mann–Whitney *U* test; **P < 0.01). **(G and H)** Super-resolution imaging (G) and FracLac analysis (H) demonstrating that SMFC microglia in DS brains are morphologically identical (amoeboid) to those in healthy brains (data, mean ± SEM; Mann–Whitney *U* test; ns, P > 0.05). Scale bars, 10 µm. **(I and J)** Super-resolution images of IBA-1 and LAMP1 immunostaining (I) and quantification of bulbous endings (J) in scattered microglia (DS, *n* = 5; healthy, *n* = 4; data, mean ± SD; Mann–Whitney *U* test; **P < 0.01). Inner insert, the LAMP1^+^ bulbous ending in the rectangle. White arrowheads, bulbous ending. Scale bars, 10 µm. **(K–N)** Super-resolution images and quantification of cleaved cas3 in scattered regions (K and L) versus the SMFC (M and N) of DS and healthy brains (DS, *n* = 5; healthy, *n* = 4; white arrowheads, apoptotic cells or debris; data, mean ± SD; Mann–Whitney *U* test; **P < 0.01). Scale bars, 10 µm. **(O)** Schematic summarizing the characteristics of the expanded SMFC in DS fetal brains compared with healthy brains.

**Figure S4.**
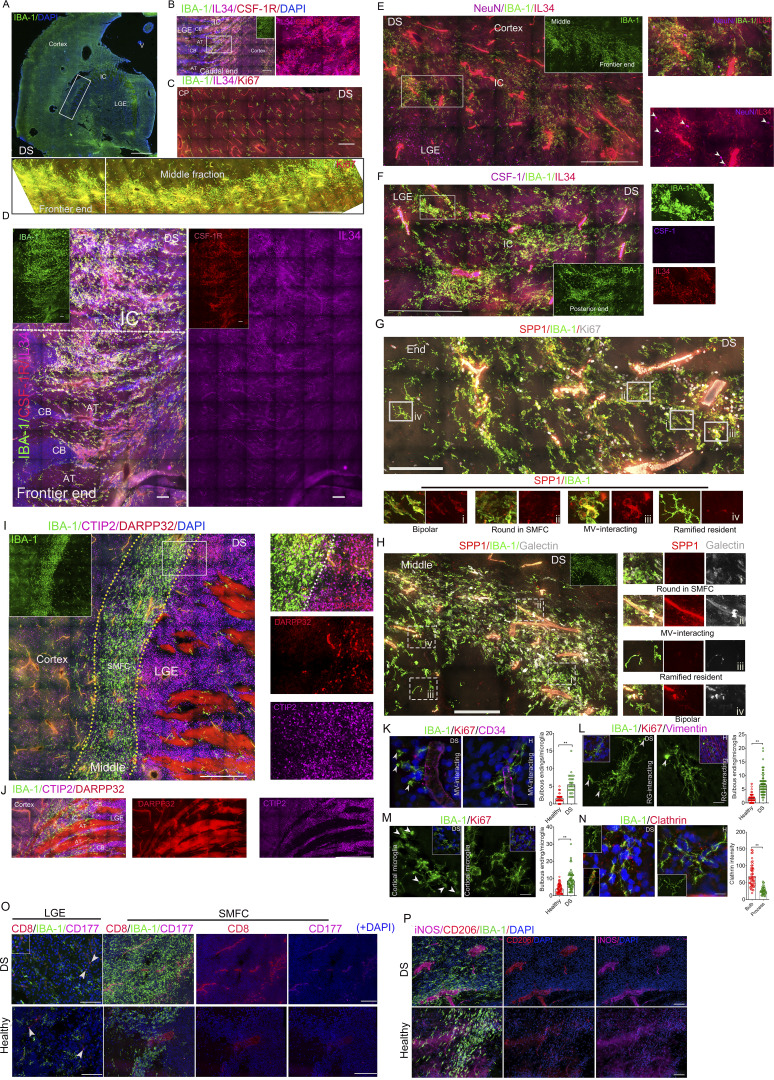
**Characterization of the SMFCs in DS fetal brains (related to Fig. 8). (A)** Whole view of a 14-gw DS fetal brain immunostained by IBA-1 and Ki67 antibodies. In the lower panel, the magnified rectangular region of the full-view image showed the entire middle section, and the other end displayed the shape and orientation of the SMFC in the DS brain. Scale bars, 2 mm (whole views) and 500 µm (below). **(B)** Stitched image of the immunostaining with IL-34, CSF-1R, and IBA-1 antibodies at one end of the SMFC of a DS fetal brain revealed that IL-34^+^ cells are preferentially enriched in the region of the SMFC characterized by a high CSF-1R level. Scale bar, 200 µm. **(C)** Immunostaining with L34, CSF-1R, and IBA-1 antibodies in a 14-gw DS fetal brain revealed that IL-34^+^ cells are almost absent in the CP. Scale bar, 200 µm. **(D)** Large SMFC region that contains a middle and an end stained by IBA-1, CSF-1R, and IL-34 antibodies in the DS fetal brain revealed that the SMFC region includes a large amount of IL-34^+^ cells. The inner insert on the right shows the IBA-1 signal, revealing IBA-1^+^ cell intensity in the SMFC. The inner insert on the left shows CSR-1R intensity in the SMFC and other regions. Scale bar, 100 µm. **(E)** Stitched image of immunostaining with IBA-1, NeuN, and IL-34 antibodies in the SMFC of the DS fetal brain (14 gw). Boxed, the magnified images (white arrowheads [bottom], NeuN^+^/IL-34^+^ cells). Scale bar, 500 µm. **(F)** Stitched image of immunostaining with IBA-1 and CSF-1 antibodies in the SMFC of the DS fetal brain. In the magnified image (below), the boxed area shows that CSF-1^+^ cells are rare in the SMFC. Scale bar, 500 µm. **(G)** SPP1, Ki67, and IBA-1 antibody immunostaining in the DS fetal brain. The magnified images: i, bipolar microglia with strong SPP1 at the end of the SMFC; ii, aggregate microglia with weak SPP1 in the middle fraction; iii, MV-interacting microglia with moderate SPP1; iv, a scattered ramified resident microglia with no SPP1. Scale bar, 200 µm. **(H)** SPP1, galectin-3, and IBA-1 antibody immunostaining in the DS fetal brain. The magnified images: i, aggregate microglia in the middle fraction with weak SPP1; ii, MV-interacting microglia with moderate SPP1; iii, ramified resident microglia with no SPP1; iv, bipolar microglia with strong SPP1. Scale bar, 200 µm. **(I)** Immunostaining of a 14-gw DS fetal brain with CTIP2, DARPP32, and IBA-1 antibodies revealed that the middle fraction of the SMFC does not overlap with the DARPP32^+^ region. Scale bar, 500 µm. **(J)** Immunostaining with CTIP2, DARPP32, and IBA-1 antibodies revealed that one end of the SMFC in the DS fetal brain overlapped with the cellular bridge of IC, whereas DARPP32^+^ did not overlap with the SMFC. AT, axon tract; CB, cellular bridge. Scale bar, 500 µm. **(K)** Super-resolution images of IBA-1, Ki67, and CD34 antibody immunostaining revealed that MV-interacting microglia in the DS fetal brain have more bulbous endings (white arrowheads, bulbous endings). Right, quantification of bulbous ending of MV-interacting microglia in DS and healthy fetal brains (DS, *n* = 3; healthy, *n* = 5; Data, mean ± SD; Mann–Whitney *U* test, **P < 0.001). Scale bar, 10 µm. **(L)** Super-resolution images of IBA-1, Ki-67, and CD34 antibody immunostaining revealed that RG-interacting microglia in DS fetal brains have more bulbous endings in the RG-interacting microglia (white arrowheads, indicated by bulbous endings). Right, bulbous ending count in the RG-interacting microglia of healthy and DS (DS, *n* = 4; healthy, *n* = 5; data, mean ± SD; Mann–Whitney *U* test, **P < 0.001). Scale bar, 10 µm. **(M)** Super-resolution images of IBA-1 and Ki67 antibody immunostaining in CP microglia in DS and healthy fetal brains. Right, bulbous ending count/microglia in the CP microglia of healthy and DS (DS, *n* = 4; healthy, *n* = 5; data, mean ± SD; Mann–Whitney *U* test, **P < 0.001). Scale bar, 10 µm. **(N)** Super-resolution images of IBA-1 and clathrin antibody immunostaining in the DS fetal brain. Right, measuring density of clathrin in bulbous endings and cellular processes (data, mean ± SD; Mann–Whitney *U* test, **P < 0.001). Scale bar, 10 µm. **(O)** Immunostaining with IBA-1, CD8, and CD177 antibodies in the healthy and DS fetal brains with the SMFC revealed that CD8^+^ T cells and CD177^+^ neutrophils are absent in the SMFCs (the inner insert in the LGE panel, intra-MV CD8^+^ T cell; white arrowheads, intra-MV CD8^+^ T cell). Scale bar, 100 µm (left) and 200 µm (right). **(P)** Immunostaining with iNOS, CD206, and IBA-1 antibodies revealed that iNOS^+^ microglia (M1) and CD206^+^ microglia (M2) are absent in the SMFC in healthy and DS fetal brains. Scale bar, 100 µm. MV, microvasculature.

To see the clinical significance of the SMFC in brain development disorders, we analyzed 14–16-gw DS fetal brains (*n* = 5). In DS fetal brains, we identified larger SMFCs with 39.0% (7,787/19,952) Ki67^+^ microglia compared with 5.58% (173/3,099) in scattered microglia (Fig. 8, A–C; Fig. S4 A; and Table S2). The DS SMFC region uniformly harbors a high density of IL-34^+^/IBA-1^−^ cells (Fig. 8, A and D; and Fig. S4, B–D), some of which express NeuN (Fig. S4 E). The microglia of the DS SMFC uniformly expressed higher CSF-1R compared with the scattered microglia (Fig. 8, E and F; and Fig. S4, B–D), and sporadically expressed CSF-1 (Fig. S4 F). Multiple SPP1^+^ bipolar and microvessel-interacting microglia exist in the SMFC margin, whereas round microglia in the SMFC and scattered ramified microglia are with weakly or not expressed SPP1 (Fig. S4, G and H). Galectin-3 is uniformly present in the microglia of the DS SMFC, including round and bipolar microglia (Fig. S4 H). Anatomically, the two ends of the DS SMFC extended into the CTIP2^+^ cellular bridge of IC, and the middle section did not overlap with the DARPP32^+^ region (Fig. 8 E; and Fig. S4, I and J). The morphology of SMFC microglia is identical to that of healthy SMFC microglia and lacks long processes, bulbous endings, and membrane ruffles (Fig. 8, G and H). Consistent with the impaired brain development of DS, more complicated processes and LAMP1^+^ bulbous endings were present in the scattered microglia (Fig. 8, I and J; and Fig. S4, K–N), and more caspase-3^+^ (cas3) cellular projections and bodies were present in the region of scattered microglia but not in the expanded DS SMFC (Fig. 8, K and L). Similar to the healthy SMFC, cas3^+^ cells, CD8^+^ cells, CD177^+^ neutrophils, iNOS^+^, and CD206^+^ microglia were absent in the DS/healthy SMFC (Fig. 8, M and N; and Fig. S4, O and P), excluding an inflammatory role of the SMFC. Together, the DS SMFC and the healthy SMFC are identical except for size (Fig. 8 O).

**Figure 9.**
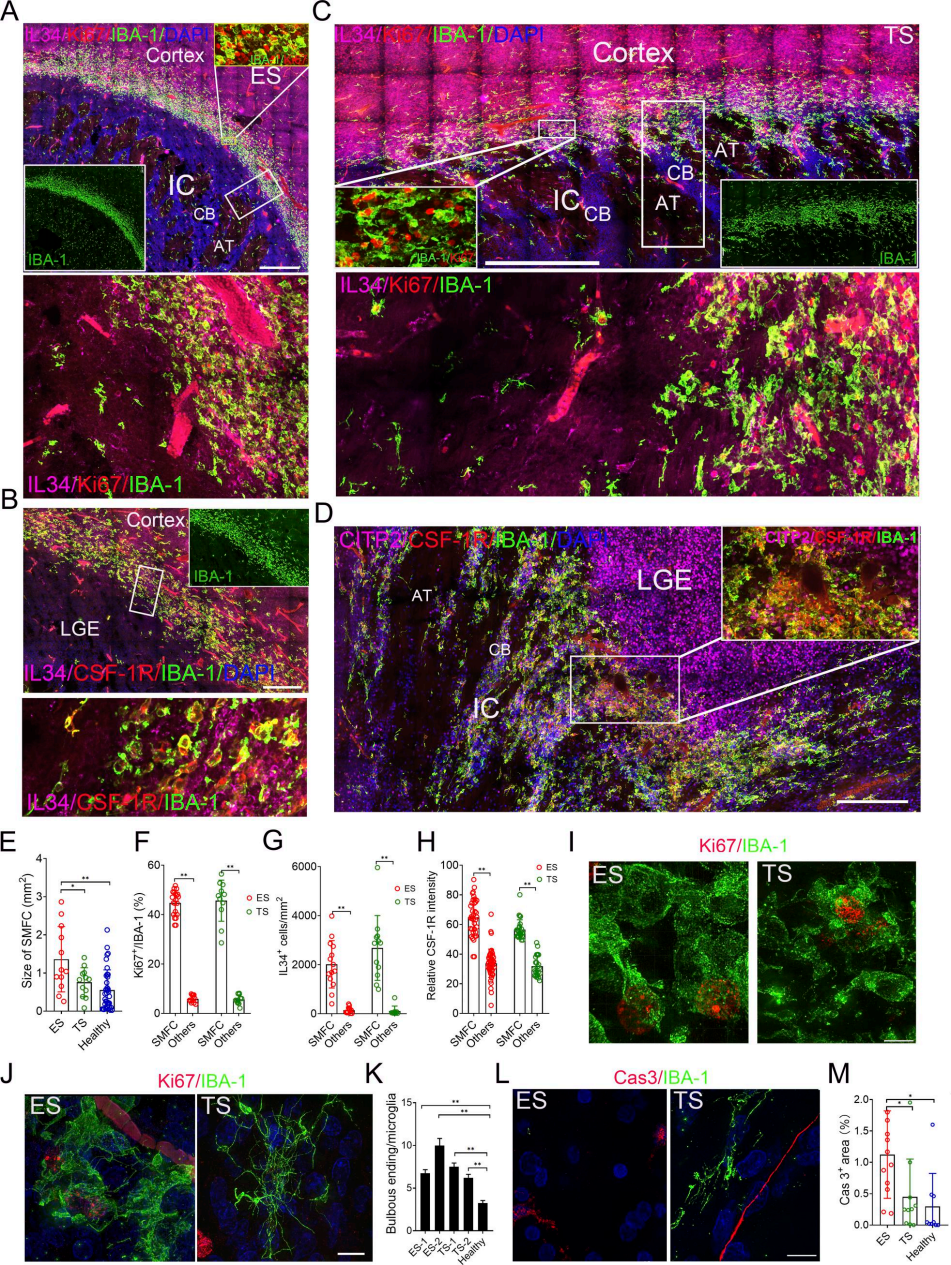
**SMFCs expand in the ES fetal brain but not in the TS fetal brains. (A)** Stitched high-resolution images of IBA-1, Ki67, and IL-34 antibody immunostaining in a 15-gw ES fetal brain reveal the total middle section of a large SMFC located in the cortical region next to the IC. The inner panel (up), typical IBA-1^+^/Ki67^+^ cells (top, without DAPI). The inner insert in the lower left shows the IBA-1^+^ cell density in the SMFC and other regions. CB, cellular bridge; AT, axon tract. Scale bar, 500 µm. **(B)** Stitched high-resolution images of IBA-1, CSF-1R, and IL-34 antibody immunostaining in a 15-gw ES fetal brain reveal that the middle section of a larger SMFC expressed strong CSF-1R. The inner insert in the lower right shows the IBA-1^+^ cell density. The lower panel shows the white rectangle from the upper panel. CB, cellular bridge; AT, axon tract. Scale bar, 100 µm. **(C)** Stitched high-resolution images of IBA-1, Ki67, and IL-34 antibody immunostaining in a TS fetal brain at 15 gw reveal the middle section of an SMFC located in the cortical region next to the IC. The inner insert in the lower left displays Ki67^+^/IBA-1^+^ cell status. The inner insert in the lower right shows the IBA-1^+^ cell density. The lower panel shows the white rectangle from the upper panel (without DAPI). Scale bar, 500 µm. **(D)** Stitched high-resolution images of IBA-1, CSF-1R, and CTIP2 antibody immunostaining in a TS fetal brain at 15 gw reveal that the end of the SMFC extended into the cellular bridge of the IC and expressed high CSF-1R (inner insert shows the rectangular region). CB, cellular bridge; AT, axon tract. Scale bar, 200 µm. **(E)** Quantification of the SMFC size per section in TS, ES, and healthy fetal brains (data are presented as the mean ± SD; one-way ANOVA; *P < 0.05, **P < 0.01). **(F–H)** Quantification of Ki67+ microglial percentage (F), IL-34^+^ cell density (G), and CSF-1R intensity (H) in the SMFC versus scattered microglia of ES and TS brains (ES, *n* = 2; TS, *n* = 2; data, mean ± SD; Mann–Whitney *U* test [F] or Sidak’s multiple comparisons test [G and H]; **P < 0.01). **(I)** Super-resolution images in ES and TS fetal brain revealed that the microglia in proliferative microglia are round and lack membrane ruffles and long processes (see also Fig. 3 A). Recording, SIM microscopy. Scale bar, 10 µm. **(J and K)** Super-resolution images (J) and quantification (K) of bulbous endings in scattered microglia of ES, TS, and healthy brains (ES, *n* = 2; TS, *n* = 2; data, mean ± SEM; one-way ANOVA with Tukey’s multiple comparisons; **P < 0.01). Scale bars, 10 µm. **(L and M)** Super-resolution images (L) and quantification (M) of cas3 cells/fibers in ES, TS, and healthy brains (ES, *n* = 2; TS, *n* = 2; data, mean ± SD; one-way ANOVA with Tukey’s multiple comparisons; *P < 0.05). Scale bars, 10 µm.

**Figure S5.**
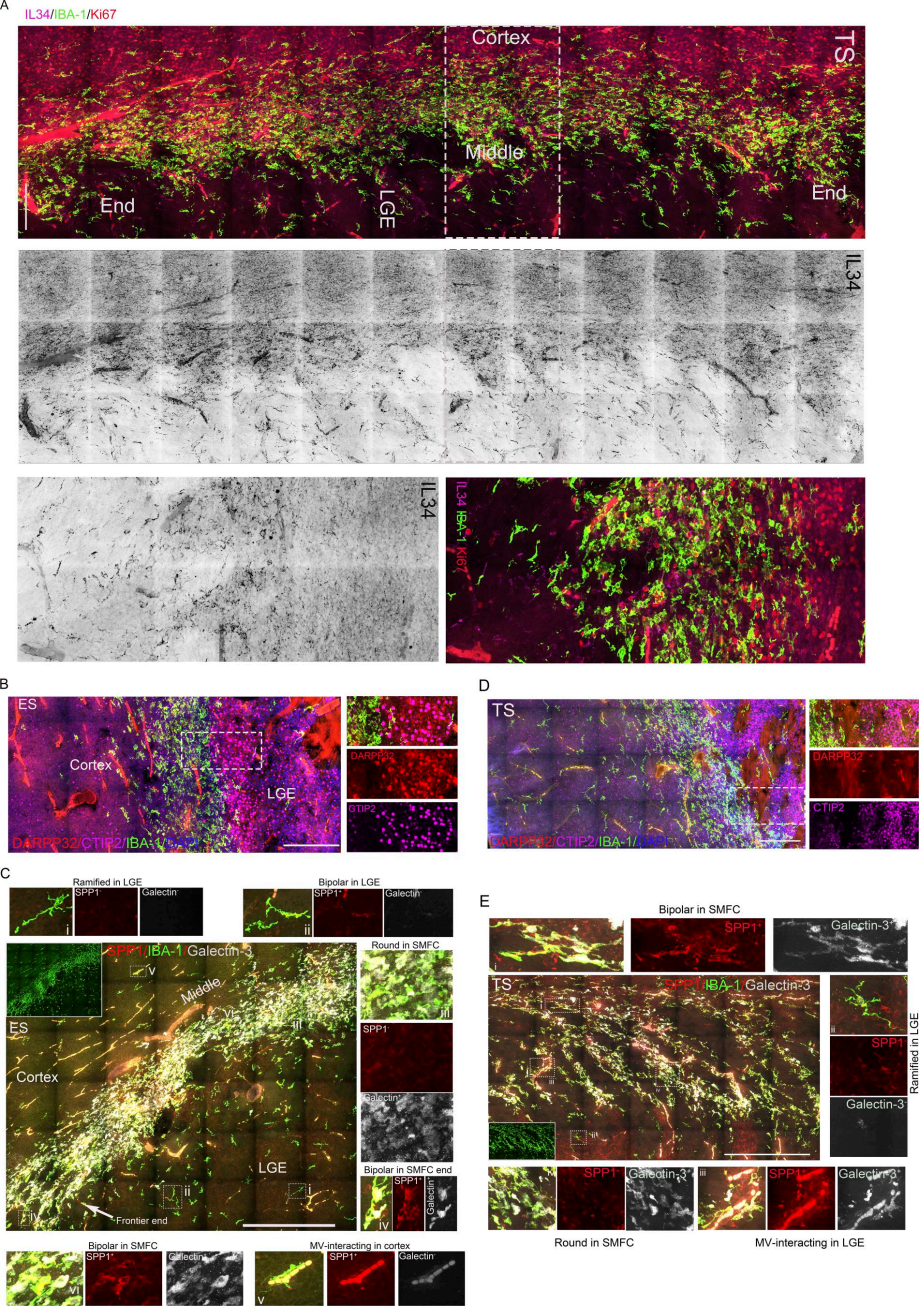
**Characterization of the SMFCs in ES and TS fetal brains (related to Fig. 9). (A)** Stitched images of the entire SMFC stained by IBA-1, Ki67, and IL-34 antibodies in the ES fetal brain revealed that IL-34^+^ cells are rare in the cortex, whereas the SMFC region contains a large amount of IL-34^+^ cells. Right, the magnified image. The middle panel shows IL-34 intensity as a black signal (inverting). The rectangles on the top and middle are a magnified image of the one on the bottom. Scale bar, 200 µm. **(B)** Stitched images of the immunostaining with CTIP2, DARPP32, and IBA-1 antibodies revealed that the middle section of the SMFC in the ES fetal brain did not overlap with the DARPP32^+^ region. The rectangles on the right show the magnified region on the left with spliced channels. Scale bar, 200 µm. **(C)** SPP1, galectin-3, and IBA-1 antibody immunostaining in the ES fetal brain showed the middle and one end section of the SMFC. The magnified images with spliced channels: i, LGE scattered ramified resident microglia with no SPP1 and no galectin; ii, LGE scattered bipolar microglia SPP1+ and weak galectin; iii, SMFC round microglia in the middle fraction with no SPP1 and weak galectin-3; iv, SMFC bipolar microglia in the end with strong SPP1 and strong galectin-3; v, cortical MV-interacting microglia with moderate SPP1 and galectin-3; vi, SMFC bipolar microglia with strong SPP1 and strong galectin-3 in the SMFC (white arrow, bipolar microglia). Scale bar, 500 µm. **(D)** Stitched images of the immunostaining with CTIP2, DARPP32, and IBA-1 antibodies revealed that the middle section of the SMFC in the TS fetal brain did not overlap with the DARPP32^+^ region. The rectangles on the right show the magnified region on the left with spliced channels. Scale bar, 200 μm. **(E)** SPP1, galectin-3, and IBA-1 immunostaining in the TS fetal brain showed the SMFC with one end section. The magnified images with spliced channels: i, SMFC bipolar microglia in the margin with strong SPP1 and strong galectin-3; ii, LGE scattered ramified resident microglia with no SPP1 and weak galectin-3; iii, MV-interacting microglia with moderate SPP1 and moderate galectin-3; iv, SMFC round microglia in the end with weak SPP1 and strong galectin-3. Scale bar, 500 µm. MV, microvasculature.

We analyzed fetal brains from individuals with ES (*n* = 2) and Turner syndrome (TS, *n* = 2) to examine SMFC changes in these conditions. ES and TS fetal brains (14–15 gw) harbor SMFCs with round microglia possessing few membrane ruffles, bulbous endings, higher CSF-1R, and a rich population of IL-34^+^/IBA-1^−^ cells (ES, 3.437 mm^2^; TS, 1.683 mm^2^) (Fig. 9, A–I; and Fig. S5 A). Ki67^+^ microglia accounted 60.5% (5,894/9,741) and 45.1% (1,251/2,771) in the TS/ES SMFCs, and 5.2% (84/1,627) and 5.4% (109/2,009) in the TS/ES scattered microglia, respectively (Fig. 9 F and Table S2). The anatomical location of the SMFC in the TS and ES brain completely overlaps with that of the SMFC in the healthy and DS fetal brain (Fig. S5, A–E). Similar to other SMFCs, the two ends of the TS/ES SMFC extended into the CTIP2^+^ cellular bridge of IC but did not overlap with the DARPP32^+^ region, and the middle region of the SMFC is located in the cortical layer next to IC (Fig. 9 D; and Fig. S5, B and D). In the ES and TS fetal brain, more complex processes with bulbous endings were present in scattered microglia (Fig. 9, J and K). Notably, identifiable cas3^+^ cells and fibers in ES but not the TS fetal brain samples are significantly higher than in the healthy brain (Fig. 9, L and M). The morphology and distribution patterns of SPP1^+^ and galectin-3^+^ cells in the ES and TS SMFC and other brain regions are identical to those of the DS and healthy fetal brains (Fig. S5, C and E). Collectively, these data highlight the SMFC as a clinically relevant structure responsive to neurodevelopmental perturbations.

## Discussion

Comprehensive structural and spatial profiling of intact microglia at super-resolution has been lacking in studies of the human fetal brain. Using super-resolution scanning of 50-µm sections, we identified novel large microglial aggregates in 12.5−22-gw human fetal brains, referred to as the SMFC. This structure is characterized by over 25% Ki67^+^ microglia, high-density IL-34^+^ cells, and evaluated CSF-1R expression and significant expansion in neurodevelopmentally impaired fetal brains, such as those with DS and ES. The microglia within the SMFC are morphologically round, lack phagocytic units and projections, and differ from well-described ramified, bipolar, and amoeboid microglia. Markedly, the appearance of the SMFC coincides with the emergence of the oSVZ in human fetal brains. While the percentage of Ki67^+^ microglia in the scattered microglia of human fetal brain is nearly identical to that of mouse fetal brain microglia (5–10%) (Barry-Carroll et al., 2023; Menassa et al., 2022), the proliferative fraction within the SMFC is over 25%. Given that the SMFC covers a substantial area of ∼7 mm^2^ in the fetal brain and exhibits robust proliferation, this structure may compensate for microglial demand during the rapid cortical expansion period.

IL-34 controls the population of murine forebrain microglia and inhibits microglial maturation (Devlin et al., 2024, *Preprint*; Kana et al., 2019). Our data—including high-density IL-34^+^ cells in the SMFC, cellular and molecular shifts in IL-34– and CSF-1–treated microglia, and IL-34 knockdown and stimulation in chimeric microglia-hCOs—support the notion that IL-34 secretion drives the SMFC formation. CSF-1R inhibition in chimeric microglia-hCOs further confirmed the necessity of the IL-34-CSF-1R pathway in this process. CSF-1R inhibition in chimeric microglia-hCOs further confirmed the necessity of the IL-34-CSF-1R pathway in this process. Neuronal death activates microglia and enhances microglial proliferation (Dang et al., 2018). Although SV40-immortalized microglia in hCOs secrete multiple cytokines, phagocytose synapses and cellular debris, and express IBA-1 and CSF-1R, their morphological complexity is significantly lower than that of iMicroglia. However, SV40 microglia form surface proliferative aggregates more quickly than iMicroglia, suggesting that they functionally resemble immature rather than mature microglia.

Similar to other observations (Seidl et al., 2001), we noticed increased neuronal death in the DS and ES fetal brains, which concomitantly present with an expanded SMFC. Trisomy of chromosome 21 in DS aberrantly activates microglia, induces neuroinflammation, and increases synaptic pruning (Jin et al., 2022; Zheng et al., 2021). However, the absence of obvious neuronal apoptosis, activated microglia with phagocytic morphology, CD8^+^ cells, and CD177^+^ neutrophils in the DS SMFC regions indicates that the expanded SMFC functions primarily as a microglial proliferation center rather than an immune response center. This hypothesis is corroborated by the finding that IL-34 enhances microglial proliferation without promoting proinflammatory or phagocytic activities. Furthermore, we observed an increased number of bulbous endings in scattered microglia across the cortex and in the LGE in 13–15-gw fetal brains. We observed an increased number of bulbous endings in scattered microglia across the cortex and in LGE in 13−15-gw fetal brains. Because the SMFC appears before the onset of high-density synaptogenesis in the fetal brain, the primary role of microglia with bulbous endings at this stage might be scavenging apoptotic cells or aberrant fibers rather than synaptic pruning.

### A limitation of this study

The precise mechanisms underlying IL-34’s role in the SMFC formation are not fully addressed in this study due to the lack of an IL-34–null ES/iPSC line to generate chimeric microglia-hCOs. Additionally, the direct relationship between the oSVZ emergence and SMFC formation remains hypothetical. Current in vitro brain organoid models do not fully recapitulate the extensive architecture of the human fetal brain, particularly the large oSVZ. Thus, validating the direct interplay between the oSVZ and the SMFC requires the development of next-generation human brain organoids with well-developed oSVZ regions. Because the oSVZ is uniquely expanded in human brain development (Lui et al., 2011), the evolutionary uniqueness of the SMFC needs to be addressed by investigating the SMFC or SMFC-like structures across different species using our approach, despite no identical structures being reported in lab species (Barry-Carroll et al., 2023; Geirsdottir et al., 2019; Lawrence et al., 2024; Yu et al., 2024).

## Materials and methods

### Human pluripotent stem cell culture

The use of hiPSCs was approved by the Ethics Committee of the Institutes of Biomedical Sciences at Fudan University, Shanghai, China (no. 28). The human embryonic stem cell line H9 (WA09) was provided by Prof. Su-Chun Zhang, Waisman Center, Madison, USA. Human pluripotent stem cells (hPSCs) were cultured under feeder-free conditions on Matrigel (354234; Corning)- or vitronectin XF–coated (07180; STEMCELL Technologies) 6-well plates in Essential 8 (E8) medium (A1517001; Gibco).

### Collection and evaluation of human fetal brain tissues

This study included two cohorts of fetal brain samples: a historical healthy cohort collected from 2006 to 2008, which was cryosectioned immediately and stored at −80°C (Table S1), and a prospective cohort collected from 2023 to 2024 (Table S1). All fetal brain tissues were obtained with informed maternal consent and institutional ethical approval (Ethics Committees of Pingdingshan Maternal and Child Health Hospital [2025-006] and Fudan University, Shanghai, China [2009-13]). Clinical diagnoses were confirmed via an Affymetrix CytoScan 750K array and karyotype analysis. Inclusion and exclusion criteria are detailed in Table S1; briefly, specimens exhibiting autolysis, internal hemorrhage, or inflammation were excluded. Gross pathology was evaluated by a senior pathologist, and tissue histopathology was assessed after immunofluorescence imaging.

### Generation of hCOs

hPSCs were dissociated into single cells and seeded into ultra-low-attachment V-bottom 96-well plates to form spheroids (day 0). On day 1, spheroids were transferred to suspension culture. From days 1 to 6, the medium consisted of 48% DMEM/F12, 48% E8 medium, 1% NEAA, 1% GlutaMAX, 1% N2 Supplement, 1% penicillin–streptomycin (P/S), 2 μM SB-431542, and 0.3 mM LDN-193189. Optionally, the WNT inhibitor XAV-939 (2.5 μM) and two SMAD inhibitors were added. From days 7 to 14, the medium was replaced with 96% Neurobasal, 1% NEAA, 1% GlutaMAX, 1% N2 Supplement, 1% P/S, 20 ng/ml EGF, and 20 ng/ml bFGF. From day 15 onward, small molecules were withdrawn, and 2% B27 was supplemented into the basal medium.

### Generation of iMicroglia/iMacrophage

The H9-GFP line was maintained on vitronectin in E8 medium. To generate embryoid bodies (EBs), cells were dissociated using Accutase (5 min, 37°C) and seeded into ultra-low-attachment 60-mm dishes in E8 medium supplemented with 10 nM Y27632 for 24 h (day 0). Hematopoietic and myeloid differentiation was induced across six stages in APEL II (05270; STEMCELL Technologies) or StemPro-34 (10639011; Gibco) media. Differentiation stages: Stage 1 (day 1, mesoderm induction): APEL II with 10 ng/ml BMP-4 and 5 ng/ml bFGF. Stage 2 (days 2–7, hematopoietic progenitors): APEL II with 10 ng/ml BMP-4, 5 ng/ml bFGF, 50 ng/ml VEGF, and 100 ng/ml SCF. Stage 3 (days 8 and 9, myeloid progenitors): APEL II with 10 ng/ml bFGF, 50 ng/ml VEGF, 50 ng/ml SCF, 10 ng/ml IGF-1, 25 ng/ml IL-3, 50 ng/ml M-CSF, and 50 ng/ml GM-CSF. Stage 4 (days 10–20, iMac precursors): 40–50 EBs were plated per well in Matrigel-coated 6-well plates in StemPro-34 supplemented with the Stage 3 cytokine cocktail. Stage 5 (days 21 and 22): suspension cells were harvested and cultured in StemPro-34 with M-CSF and GM-CSF increased to 100 ng/ml. Stage 6 (days 23–26): StemPro-34 with 5 ng/ml bFGF, 50 ng/ml VEGF, 50 ng/ml SCF, 10 ng/ml IGF-1, 100 ng/ml M-CSF, and 100 ng/ml GM-CSF.

### Engineering of chimeric microglia-hCOs

We adapted a previously established protocol to generate microglia–cortical organoids. Briefly, 1 × 10^5^ day-26 iMacs were cocultured with one day-26 hCO in 1 ml of organoid medium supplemented with 100 ng/ml M-CSF and 20 ng/ml IL-34 in an ultra-low-attachment 3.5-cm dish. On day 2, an additional 1 ml of supplemented organoid medium was added. From day 5, half of the medium was changed every 3 days for 18 days.

### Human immortalized microglial (SV40) culture and SV40-hCO generation

SV40-immortalized microglia were purchased from Applied Biological Materials Inc. (T3961). Cells were maintained in Prigrow III (TM003) or DMEM/F12 with 10% FBS on Applied Cell Extracellular Matrix–coated plates, and passaged at 80% confluence using 0.25% trypsin. To generate SV40-hCOs, SV40 microglia were introduced into the hCO culture medium on days 30 and 60 at a density of 1 × 10^4^ cells/ml. The coculture was incubated overnight on an orbital shaker to facilitate microglial integration. The medium was subsequently replaced with microglia-free medium and maintained for 7 days.

### High-density microelectrode array electrophysiology

HD-MEA chips (MaxLab) were coated with 0.07% (vol/vol) poly(ethylenimine) (Sigma-Aldrich) and laminin. Whole hCOs were mounted onto the chips and secured with a droplet of Matrigel. Following a 30-min gelation period at 37°C, the chips were filled with BrainPhys Neuronal Medium (STEMCELL Technologies) supplemented with 2% NeuroCult SM1, 1% N2 Supplement-A, 1× GlutaMAX, 20 ng/ml BDNF, 20 ng/ml GDNF, 20 ng/ml NT-3, 1 mM dibutyryl-cAMP, and 1× Antibiotic–Antimycotic. Recordings commenced at least 7 days after plating. Spontaneous activity was mapped sequentially across the 1,020-electrode array (30 s/configuration) at a 20-kHz sampling rate. Spikes were detected utilizing a threshold of 5× the root-mean-square noise of the band-pass–filtered signal. Data reflect recordings from 3 hCOs and three SV40-hCOs.

### Organoid slice preparation and lentiviral transduction

Day-37 hCOs were embedded in 3% low-melting agarose and sectioned into 300-μm slices using a vibratome (WPI). Slices were recovered in ultra-low-attachment dishes for 5 days. For IL-34 knockdown, slices were incubated for 24 h in 96-well plates containing IL-34 lentivirus (1:10 and 1:100 dilutions) and 5 μg/ml Polybrene. After transduction, slices were recovered in fresh medium for 48 h before 2 × 10^4^ SV40 microglia were seeded per well (day 0). Cocultures were maintained on an orbital shaker, with medium replaced on days 1 and 3, and fixed in 4% paraformaldehyde (PFA) on day 5.

### In vitro cytokine treatments and EdU assays

For cytokine stimulation, SV40 microglia or hCOs were treated with 20 or 100 ng/ml of human recombinant IL-34 (HumanKine, HZ-1316), CSF-1, or IFN-γ for up to 120 h. For proliferation assays, EdU (10 µM; Invitrogen) was added to the culture medium for 12 h prior to fixation. Cells/organoids were permeabilized and processed utilizing the Click-iT reaction kit according to the manufacturer’s protocol (Chen et al., 2022).

### Histology, immunostaining, and image acquisition

Organoids and fetal brain samples were fixed in 4% PFA for 30 min or 24 h, respectively, cryoprotected in 30% sucrose, and embedded in OCT. Sections (15 µm for organoids; 50 µm for fetal brains) were blocked and permeabilized in 0.3% Triton X-100/10% normal donkey serum for 1 h, followed by overnight incubation at 4°C with primary antibodies (Tables S3 and S4). Slices were washed and incubated with fluorophore-conjugated secondary antibodies (Table S5) and DAPI for 1 h at room temperature. High-resolution imaging was performed using an Olympus SpinSR10 spinning disk confocal microscope, a Nikon Structured Illumination Microscope(∼64-nm resolution), a Leica wide-field confocal microscope, or a Zeiss 880 laser scanning confocal microscope.

### RNA-seq and transcriptomic analysis

Bulk RNA-seq was performed on SV40 microglia treated with 20 ng/ml IFN-γ, 20 ng/ml IL-34, 20 ng/ml CSF-1, or 100 ng/ml CSF-1 (*n* = 3 biological replicates per condition), alongside unstimulated controls (*n* = 6). Libraries were sequenced on an Illumina NovaSeq 6000 platform (PE150 strategy). Raw reads were quality-filtered, and differential gene expression analysis was executed using the DESeq2 package in R (version 4.4.1). Differentially expressed genes (DEGs) were defined by an absolute log_2_ fold change ≥1.0 and a Benjamini–Hochberg adjusted P <0.05. Gene Ontology functional enrichment analysis for DEGs was performed using the clusterProfiler package, assessing Biological Process, Molecular Function, and Cellular Component categories.

### Image analysis and statistics

Cell quantification, fluorescence intensity, and spatial distributions were analyzed using ImageJ (Fiji) and Imaris 9.8. For microglial morphometry, ImageJ was used to binarize and skeletonize single-cell images, followed by the “Analyze Skeleton” plugin. FracLac analysis was employed to determine cellular complexity (box-counting method). Statistical analyses were performed using GraphPad Prism. Data are presented as the mean ± SD or SEM, as indicated. Two-group comparisons were assessed using the Mann–Whitney *U* test or Student’s *t* test, while multiple comparisons were analyzed via one-way ANOVA followed by Tukey’s or Holm–Sidak’s post hoc tests. Significance was defined as P ≤ 0.05.

### Online supplemental material

Table S1 shows baseline characteristics of fetal brain samples. Table S2 shows characteristics of the SMFC in healthy and diseased fetal brain samples. Table S3 shows antibodies for immunostaining with their purpose. Table S4 shows the list of primary antibodies. Table S5 shows the list of secondary antibodies, dyes, and reagents. Fig. S1 shows the changes of microglial density and complexity in the fetal brains (related to Fig. 1). Fig. S2 shows the proliferative microglial aggregates are present in the same location next to the LGE, absent in the LGE and ventricle of human fetal brains with different gw, and harbor SPP1^+^ bipolar and microvasculature-interacting microglia on the border region (related to Figs. 2, 3, 4, and 5). Fig. S3 shows characterization of hCOs and chimeric iMicroglia-hCOs (related to Fig. 6). Fig. S4 shows characterization of the SMFCs in DS fetal brains (related to Fig. 8). Fig. S5 shows characterization of the SMFCs in ES and TS fetal brain (related to Fig. 9).

## Supplementary Material

Table S1 shows baseline characteristics of fetal brain samples.

Table S2 shows characteristics of the SMFC in healthy and diseased fetal brain samples.

Table S3 shows antibodies for immunostaining with their purpose.

Table S4 shows the list of primary antibodies.

Table S5 shows the list of secondary antibodies, dyes, and reagents.

## Data Availability

All relevant data are included in the paper and are available upon reasonable request. The bulk RNA-seq and proteomic datasets generated and/or analyzed during this study are available in the Sequence Read Archive under the following accession numbers: PRJNA1152524, PRJNA1156058; and the National Genomics Data Center BioProject database under the following accession number: PRJCA057062.
