# Supplementary material for: Identification of secondary microglial formation centers in the human fetal brain
Source: J Exp Med. 2026 May 18;223(6):e20251801. doi: 10.1084/jem.20251801 (PMC13182777; doi:10.1084/jem.20251801)
Supplement: Table S3 — shows antibodies for immunostaining with their purpose. [file jem_20251801_tables3.docx]

**Table S3: Antibodies for immunostaining with their purpose.**

| **Name** | **Purpose** | **Figure** |
| --- | --- | --- |
| **General markers in developing human brain** | | |
| SOX2 | Neural progenitor marker | F6 |
| Ki67 | Proliferative cells marker | F1, F2, F4-6, F8-9, S2, S3, S5 |
| PH3 | Proliferative cells marker | S3 |
| TUJ1 | Neuron marker | S3 |
| NEUN | Mature neurons marker | F6, S4 |
| PSD95 | Post-synaptic proteins marker | S3 |
| GFAP | Astrocyte marker | F1 |
| Vimentin | Radial glia marker | F1, F2, F4 |
| IBA-1 | Microglia marker | F1-6, F8-9 |
|  |  | S1-S5 |
| CD34 | Microvessel marker | F1, F2, S1, |
| PAX6 | Forebrain markers | S1 |
| OTX2 | Forebrain/Midbrain marker | S1 |
| **Region-specific markers in developing human brain** | | |
| Cortex |  |  |
| PAX6 | Dorsal forebrain progenitor marker | S1, S3, S4 |
| TBR2 | Intermediate progenitor marker | S3 |
| CTIP2 | One of cortical layer neuron markers | F3, F8, F9, S3, |
| LGE |  |  |
| CTIP2 | Striatal MSN marker | S4, S5 |
| DARPP32 | Striatal MSN marker | S4, S5 |
| **Microglia markers and Immune cell markers** | | |
| SPP1 | Migratory microglia | F4, S2, S4, S5 |
| Galectin-3 | Microglia | F4, S2, S5 |
