# Supplementary material for: Identification of secondary microglial formation centers in the human fetal brain
Source: J Exp Med. 2026 May 18;223(6):e20251801. doi: 10.1084/jem.20251801 (PMC13182777; doi:10.1084/jem.20251801)
Supplement: Table S4 — shows the list of primary antibodies. [file jem_20251801_tables4.docx]

**Table S4. Primary antibodies list**

| **Antibody** | **Host** | **Cat.** | **Concentration** |
| --- | --- | --- | --- |
| Caspase3 | Rabbit | #9669 | 1:1000 |
| CD177 | Mouse | MEM166 | 1:1000 |
| CD206 | Mouse | 321102 | 1:500 |
| CD34 | Mouse | ab8536 | 1:1000 |
| CD8 | Rabbit | ab4505 | 1:1000 |
| Clathrin | Rabbit | ab172958 | 1:500 |
| CSF-1R | Rabbit | ab313648 | 1:500 |
| CTIP2 | Rat | ab18465 | 1:1000 |
| DARPP32 | Rabbit | ab1656 | 1:1000 |
| GFAP | Mouse | ab279290 | 1:1000 |
| GFAP | Rabbit | Z0334 | 1:1000 |
| GFP | Chicken | ab6556-25 | 1:1000 |
| GSH2 | Rabbit | 3388451 | 1:500 |
| IBA-1 | Goat | ab5076 | 1:1000 |
| IL34 | Mouse | ab101443 | 1:500 |
| iNOS | Rabbit | ab178945 | 1:500 |
| Ki67 | Rabbit | LV1825852 | 1:1000 |
| MAP2 | Mouse | m1406 | 1:1000 |
| MAP2 | Rabbit | sc20172 | 1:5000 |
| MASH1 | Mouse | 556604 | 1:500 |
| MEIS1/2 | Goat | sc-10599 | 1:500 |
| OTX2 | Goat | AF1979 | 1:1000 |
| PAX6 | Rabbit | 901301 | 1:1000 |
| PH3 | Mouse | 9706 | 1:1000 |
| PSD95 | Rabbit | ab18258 | 1:1000 |
| SOX2 | Mouse | MAB2018 | 1:1000 |
| TBR2 | Sheep | AF6166 | 1:500 |
| TUJ1 | Mouse | T8660 | 1:1000 |
| TUJ1 | Rabbit | PRB-435P | 1:10000 |
| Vimentin | Mouse | 5G3F10 | 1:1000 |
| GM-CSF1 | Rabbit | Ab316862 | 1:200 |
| LAMP1 | Mouse | H4A3 | 1:1000 |
| **SPP1** | **Mouse** | **HY-P990117** | **1:1000** |
| **Galectin-3** | **Rabbit** | **87985** | **1:1000** |
| CD14 | Mouse | 567731 | Flow cytometry |
| CD11b | Mouse | ICRF44 | Flow cytometry |
| CD86 | Mouse | 560958 | Flow cytometry |
| CD163 | Mouse | 568186 | Flow cytometry |
