# Supplementary material for: Identification of secondary microglial formation centers in the human fetal brain
Source: J Exp Med. 2026 May 18;223(6):e20251801. doi: 10.1084/jem.20251801 (PMC13182777; doi:10.1084/jem.20251801)
Supplement: Table S5 — shows the list of secondary antibodies, dyes, and reagents. [file jem_20251801_tables5.docx]

**Table S5. Secondary antibodies, dyes, and reagent list**

| Antibody | company | concentration |
| --- | --- | --- |
| Alexa Fluor 488 Donkey anti-mouse IgG | Invitrogen | 1:1000 |
| Alexa Fluor 594 Donkey anti-mouse IgG | Invitrogen | 1:1000 |
| Alexa Fluor 594 Donkey anti-rabbit IgG | Invitrogen | 1:1000 |
| Alexa Fluor 488 Donkey anti-rabbit IgG | Invitrogen | 1:1000 |
| Alexa Fluor 594 Donkey anti-goat IgG | Invitrogen | 1:1000 |
| Alexa Fluor 488 Donkey anti-rat IgG | Invitrogen | 1:1000 |
| Cy5 AffiniPure Donkey Anti-Goat IgG (H+L) | Jackson | 1:500 |
| Cy5 AffiniPure Donkey Anti-mouse IgG (H+L) | Jackson | 1:500 |
| Cy5 AffiniPure Donkey Anti-rabbit IgG (H+L) | Jackson | 1:500 |
| DAPI | Sigma | 1:1000 |
| EdU assay | APExBio |  |
| PLX5622 | APExBio |  |
| IL34 shRNA (h) lentiviral particles | Santa Cruz | Sc-92990-V |
| IL-34 PLUS | Okine | Qk091 |
